# NNT‐induced tumor cell “slimming” reverses the pro‐carcinogenesis effect of HIF2a in tumors

**DOI:** 10.1002/ctm2.264

**Published:** 2021-01-12

**Authors:** Zhiyong Xiong, Wei Xiong, Wen Xiao, Changfei Yuan, Jian Shi, Yu Huang, Cheng Wang, Xiangui Meng, Zhixian Chen, Hongmei Yang, Ke Chen, Xiaoping Zhang

**Affiliations:** ^1^ Department of Urology Union Hospital, Tongji Medical College Huazhong University of Science and Technology Wuhan China; ^2^ Institute of Urology Union Hospital, Tongji Medical College Huazhong University of Science and Technology Wuhan China; ^3^ Department of Pathogenic Biology School of Basic Medicine Huazhong University of Science and Technology Wuhan China; ^4^ Department of Nephrology Union Hospital Tongji Medical College Huazhong University of Science and Technology Wuhan China

**Keywords:** clear cell renal cell carcinoma (ccRCC), HIF2a, nicotinamide nucleotide transhydrogenase (NNT), pro‐carcinogenesis effect, tumor cell “slimming”

## Abstract

**Background:**

HIF2a and lipid accumulation play key roles in the progression of clear cell renal cell carcinoma (ccRCC). Tumor cell “slimming” is a new concept in which tumor cells with abnormal lipids efficiently consume lipids to inhibit tumor progression without producing additional ATP. However, their respective regulatory mechanisms are still unclear. The purpose of this study is uncovering the links between these three key elements of ccRCC to elucidate new mechanisms of ccRCC metabolic abnormalities and providing a basis for new drug development for ccRCC.

**Methods:**

Bioinformatics screening and analyses were performed in ccRCC according to TCGA‐KIRC database. qRT‐PCR, luciferase reporter assay, western blot, chromatin immunoprecipitation (ChIP) assays, and other biological methods were used to explore and verify related pathways. Various cell line models and animal models were used to perform related functional experiments.

**Results:**

Screening based on sequencing data after HIF2a knockdown and three independent mitochondrial metabolism‐related gene sets showed that nicotinamide nucleotide transhydrogenase (NNT) was a mediator between HIF2a and tumor cells “slimming.” Further research showed that NNT had significant prognostic predictive value and was downregulated in ccRCC. It is regulated by HIF2a and can significantly activate lipid browning‐mediated tumor cell “slimming.” Mechanistic investigations indicated that HIF2a enhanced the expression of miR‐455‐5p via binding to HIF2a‐related response elements in the miR‐455‐5p promoter, which suppresses NNT expression by binding to its 3′ untranslated region.

**Conclusions:**

Our study revealed a novel mechanism by which HIF2a decreased NNT level through a microRNA that suppressed tumor cell “slimming,” resulting in the progression of ccRCC. This mechanism provides a fresh perspective of lipid accumulation in ccRCC and may help target novel strategies for the treatment of tumors with abnormal lipid metabolism.

## Graphical abstract

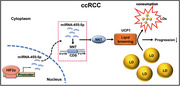

**Abbreviations**
ATP: Adenosine triphosphate
CT: Cycle threshold
DMEM: dulbecco's modified eagle medium
EDTA: Ethylene Diamine Tetraacetic Acid
NADH: Nicotinamide adenine dinucleotide
NADPH: nicotinamide adenine dinucleotide phosphate
PBS: phosphate buffer saline
PBST: Phosphate Buffered Saline with Tween 20
PCR: Polymerase Chain Reaction
PMSF: Phenylmethanesulfonyl fluoride
RIPA: Radio Immunoprecipitation Assay
UCP1: uncoupling protein‐1

## 1 INTRODUCTION

Renal cell carcinoma (RCC) is one of the most common malignant tumors of the urinary system.^1^ And the most common pathological type is clear‐cell RCC (ccRCC).^2^ Previous studies on ccRCC indicated that the constitutive hypoxia inducible factor (HIF) activation and an abundance of intracellular lipid droplets (LDs) were the most important recognized hallmarks of ccRCC.^3^, ^4^ The pathogenesis of most ccRCC tumors is characterized by the activation of HIF caused by the loss of von Hippel‐Lindau.^5^ HIF1a and HIF2a are two subunits of HIFa which regulate HIF‐mediated processes. Nowadays, HIF1a is considered as a tumor suppressor that inhibits tumor growth, while HIF2a generally acts as an oncogene that directly promotes cell proliferation and metabolic alterations.^6^, ^7^, ^8^, ^9^ Therefore, HIF2a and its related pathways are a focus of current ccRCC research.^10^

Abnormal LD accumulation is also a significant feature of ccRCC, which is mainly composed of triglycerides and cholesterol esters.^11^ This abnormal lipid accumulation plays pivotal roles in ccRCC. It stabilizes the endoplasmic reticulum to promote tumor progression. The study also found that these abnormal lipids were highly correlated with HIF2a, and the HIF2a‐associated pathway was critical for the accumulation of lipids in ccRCC. Therefore, this abnormal lipid accumulation is also known as HIF2a‐dependent lipid storage.^12^ Nevertheless, the mechanisms responsible for the dispersal of HIF2a‐dependent lipid storage while simultaneously reversing the pro‐carcinogenesis effect of HIF2a on ccRCC remain unclear.

Human adipose tissue consists of white adipose tissue (WAT) for energy storage and brown adipose tissue (BAT) for heat generation.^13^ The process of converting WAT to BAT is called lipid browning, by which the lipids are converted from an energy storage state to a heat‐producing state.^13^, ^14^ This is an interesting process in which lipids are not only consumed, but also do not produce additional ATP, and it is mainly directly mediated by UCP1, the most important marker of BAT, and localized in mitochondria.^15^ Traditionally, lipid browning has extensively carried out research in the field of diabetes and obesity, but it has not been involved in the field of tumors. Our previous research focused on the transformation of lipid browning in tumors. Studies have found that similar reactions can occur in tumors, which can lead to a significant consumption of LDs in tumor cells, resulting in the significant inhibition of tumor progression. This efficient way to consume lipids and inhibit tumor progression was first discovered and defined as tumor cell “slimming.”^16^ However, the regulation and activation of tumor cell “slimming” remains unclear currently.

Nicotinamide nucleotide transhydrogenase (NNT) is located in the inner mitochondrial membrane which is encoded by a highly conserved gene.^17^ It plays a vital role in regulating the dynamic balance between NADH and NADPH in mitochondria.^18^ NADH and NADPH which act as electron carriers are critical co‐enzymes in processes related to metabolism, including energy biosynthesis, transduction, and redox homeostasis.^19^, ^20^ Previous studies showed that NNT was highly associated with various metabolic processes. It had been reported that NNT could regulate the tricarboxylic acid (TCA) cycle, glucose metabolism, glutamine metabolism, lipid metabolism, oxidative stress, and the balance of the redox.^21^, ^22^, ^23^ However, very few studies have been done on the roles of NNT in tumors, and especially it has never been reported in ccRCC.

Our study unveiled a new mechanism by which HIF2a regulated lipid metabolism and promoted tumor progression in ccRCC; in this mechanism, HIF2a inhibits NNT expression via miR‐455‐5p to promote lipid accumulation, resulting in the progression of tumor. Moreover, the restoration of NNT significantly promotes lipid browning resulting in tumor cell “slimming” to reduce the abnormal lipid accumulation, leading to the repression of pro‐carcinogenesis effects of HIF2a. These findings provide a fresh perspective of ccRCC and may help target innovative strategies for the treatment of tumors.

## 2 MATERIALS AND METHODS

### 2.1 Cell culture and reagents

Five RCC cell lines (786‐0, A498, ACHN, CAKI, and OSRC) and the control cell line HEK‐293 were obtained from The American Type Culture Collection (ATCC, Manassas, VA). Ten percent fetal bovine serum and 1% penicillin‐streptomycin were added to DMEM high glucose medium for cell culture and incubator set in a 5% CO2 at 37°C. The mycoplasma negative certificates of the cells are shown in supporting information 1 and 15.

NNT‐specific overexpression lentivirus is obtained from Genechem, China. The shRNA for NNT and HIF2a is also purchased from Genechem, China. The mimics/inhibitors for miR‐455‐5p are all purchased from Guangzhou Ribobio, China. Oil red dye is obtained from Wuhan Servicebio technology. Triglyceride assay kit (A110‐1‐1) is provided by Nanjing Jiancheng Bioengineering Institute. Mycoplasma PCR Detection Kit (C0301S) and Mycoplasma Stain Assay Kit (C0296) are bought from Beyotime Biotechnology.

### 2.2 Mycoplasma detection

PCR mycoplasma detection: According to the instructions of Mycoplasma PCR Detection Kit (C0301S), the cell culture supernatant and cell DNA are detected, respectively. First, perform the 1st PCR reaction, mix ultrapure water, DNA sample, 1st PCR Primer Mix (50X), and Easy‐Load PCR Master Mix (Green, 2X) according to the ratio. We follow STEP1 94°C 30 seconds, STEP2 94°C 30 seconds, STEP3 55°C 2 minutes, STEP4 72°C 1 minute, STEP5 Go to STEP2 for 30‐35 cycles, STEP6 72°C 5 minutes, STEP7 4°C forever for PCR reaction. Next, perform 2nd PCR reaction, mixing ultrapure water, 1st PCR product, 2nd PCR Primer Mix (50X), Easy‐Load PCR Master Mix (Green, 2X) in proportions. Perform PCR under STEP1 94°C 30 seconds, STEP2 94°C 30 seconds, STEP3 55°C 2 minutes, STEP4 72°C 1 minute, STEP5 Go to STEP2 for 30 cycles, STEP6 72°C 5 minutes, STEP7 4°C forever. Perform agarose gel electrophoresis on the products of 1st PCR and 2nd PCR in the corresponding order.

Mycoplasma stain assay: According to the instructions of Mycoplasma Stain Assay Kit (C0296), we cultivate cells in a six‐well plate to 50‐80% confluence, dilute Hoechst staining solution 1:10 with PBS (the concentration is 10%), fix with appropriate amount of fixative for 10‐20 minutes, remove fixative, dry in the air, add an appropriate amount of 10‐fold dilution stain with Hoechst staining solution at room temperature for 10‐30 minutes, remove the staining solution, dry in the air, add dropwise the anti‐fluorescence quenching mounting solution provided in the kit and observe the blue fluorescence under a fluorescence microscope after mounting.

**Highlights**

- It is the first time to clarify the regulatory relationship between HIF2a and tumor “slimming.”
- HIF2a decreases the NNT level through miR‐455‐5p that suppresses tumor “slimming,” resulting in ccRCC progression.
- This mechanism provides a fresh perspective of ccRCC and may help target novel strategies for the treatment of tumors with abnormal lipid metabolism.

### 2.3 Whole transcriptome sequencing

The whole transcriptome sequencing after HIF2α stable knockdown is supported by Oebiotech, China, and the contract number is OE2017H0149S. After extracting RNA, we used the mirVana miRNA Isolation Kit (Ambion) using the Agilent 2100 Bioanalyzer (Agilent Technologies, Santa Clara, CA) to evaluate RNA integrity to obtain samples with RNA integrity number (RIN) ≥ 7. Subsequently, to construct the gene library, we chose the TruSeq Stranded mRNA LTSample Prep Kit from Illumina, San Diego, CA. Finally, for sequencing analysis, we used the Illumina sequencing platform (HiSeqTM 2500 or Illumina HiSeq X Ten) to obtain 125 bp/150 bp paired‐end reads.

### 2.4 Lentivirus construction

The restriction enzymes are used to prepare the linearized vector, and the target fragment is prepared by annealed primers. The designed primers add restriction sites at both ends. After annealing, the primer contains the same restriction sites as the two ends of the linearized cloning vector. The linearized vector and the annealing product are used to prepare the reaction system, carry out the ligation reaction, and the product is directly transformed. Use PCR to identify individual clones and use sequencing for analysis. The correct cloned bacterial liquid is expanded, cultured. Subsequently, the high‐purity plasmid is extracted for lentivirus packaging. The tool vector plasmid with target sequence or target gene, the helper plasmid 1.0 for lentivirus packaging, and the helper plasmid 2.0 for lentivirus packaging are co‐transfected into 293T cells. The virus is harvested 48‐72 hours after the transfection is completed. Finally, the lentivirus is concentrated and purified for preservation.

**Table:** **Table A**: Lentivirus construction information

Name	Gene ID	Genebank	Vector information
NNT	23530	NM_012343	Ubi‐MCS‐3FLAG‐CBh‐gcGFP‐IRES‐puromycin, GV492
NNT‐RNAi	23530	NM_182977	hU6‐MCS‐CBh‐gcGFP‐IRES‐puromycin, GV493
HIF2a‐RNAi	2034	NM_001430	hU6‐MCS‐Ubiquitin‐EGFP‐IRES‐puromycin, GV248

### 2.5 Lentiviral transduction

Culture the cells in a 6‐well plate to a confluency of 30%, and change the medium to a serum‐free medium. Add 40 uL of transfection reagent A (Genechem, China), transfection reagent P (Genechem, China) and 40 moi lentivirus for virus transfection. After 8 hours, change to a serum‐enriched medium to culture the above cells. Then cells were cultured for 3 days, and puromycin was added for stable cell line selection. Expression verification after 3 days of continuous puromycin treatment.

### 2.6 Mimic/inhibitor construction

Mimic refers to miRNA mimics, which are double‐stranded sequences that are artificially synthesized to simulate endogenous miRNAs. Inhibitor refers to miRNA inhibitor, which is a single‐stranded sequence that uses artificial chemical synthesis to specifically inhibit target miRNA. All miRNA mimic and inhibitor design sequence information is derived from miRNA general database, miRbase (http://www.mirbase.org/). The detailed technical solution is provided by Guangzhou Ribobio, China. The related sequence information of miR‐455‐5p is as follows: mimic: 5′‐UAUGUGCCUUUGGACUACAUCG ‐3′ / 3′‐AUACACGGAAACCUGAUGUAGC‐5′; inhibitor: 5′‐CGAUGUAGUCCAAAGGCACAUA‐3′.

### 2.7 Tissue samples

Forty‐eight pairs of human ccRCC tissues and paired adjacent tissues (at least 5 centimeters from the tumor tissue) were provided by the Department of Urology, Union Hospital, Tongji Medical College (Wuhan, China). Tissue specimens were preserved in liquid nitrogen. The content of the experiment was fully informed by the patients. And the Huazhong University of Science and Technology Institutional Review Board authorized this study.

### 2.8 Immunohistochemistry and immunofluorescence staining

The tissue specimens were cut into 4 μm paraffin‐embedded sections. EDTA was applied to deparaffinize, rehydrate. The conditions used for antigen retrieval were 120°C incubation for 5 minutes. Then, treat with 3% H2O2 for 15 minutes at room temperature. Subsequently, after the serum‐blocking was completed, we used primary antibodies to incubate the tissue sections overnight at 4°C. At room temperature, immunodetection was performed using secondary antibody. Finally, use DAB to visualize the results. Then, hematoxylin was used to counterstain the sections. The primary antibody used in immunohistochemistry (IHC) is the same as the corresponding antibody involved in western blot. The relevant antibodies information was shown as follows: NNT (Abclonal, A4561), PGC1A (Abclonal, A12348), UCP1 (Abcam, ab10983), DIO2 (Proteintech, 26513‐1‐AP), CIDEA (Santa Cruz, sc‐8730‐R), and Ki67 (Abclonal, A2094).

When it comes to immunofluorescent staining, the relevant specimens were first fixed with 4% paraformaldehyde using TritonX to permeabilize for 10 minutes. At the same time, 5% goat serum is used to block the above samples. Subsequently, we used primary antibodies to incubate the cells. Finally, the secondary antibody from Abclonal (AS069) was used. And nuclei were stained with the DAPI obtained from SIGMA. The primary antibody used for immunofluorescence is the same as the corresponding antibody involved in western blot. The relevant antibodies information was shown as follows: UCP1 (Abcam, ab10983).

For observing the LDs, we used 1 mg/mL of Bodipy 493 of 503 dye from Invitrogen to display LDs. The result image was taken by using a confocal microscope.

### 2.9 RNA isolation and real‐time PCR analysis

The sample RNA was extracted using TRizol reagent (Thermo Fisher Scientific, Waltham, MA). Subsequently, the concentration and purity of the extracted RNA were tested by the NanoDrop 2000 spectrophotometer (NanoDrop Technologies, Wilmington, DE). We took 1 microgram of the above product for reverse transcription process and used the SYBR Green mix (YEASEN, China) to perform PCR by StepOnePlus Real‐Time PCR System (Thermo Fisher Scientific). MicroRNAs primers were obtained from RiboBio, and gene primers are as follows:

**Table:** **Table B**: List of primers for PCR

Primer	Sequence
**GAPDH**	
Forward	5′‐GAGTCAACGGATTTGGTCGT‐3′
Reverse	5′‐GACAAGCTTCCCGTTCTCAG‐3
**NNT**	
Forward	5′‐ GTCTCCTGAAATCTGCCCCT ‐3′
Reverse	5′‐ CAGCACAGTGATAACGACGG ‐3′
**PGC1A**	
Forward	5′‐ AGCCTCTTTGCCCAGATCTT ‐3′
Reverse	5′‐ GGCAATCCGTCTTCATCCAC ‐3′
**UCP1**	
Forward	5′‐ GCGGATGAAACTCTACAGCG ‐3′
Reverse	5′‐ TTGATTCCGTGGAGATGGCT ‐3′
**CIDEA**	
Forward	5′‐ TCTGGTGCTGGAGGAAGATG ‐3′
Reverse	5′‐ TGACTCTCGCTATTCCCGAC‐3′
**DIO2**	
Forward	5′‐ TCTCCAACTGCCTCTTCCTG ‐3′
Reverse	5′‐ ACCATTGCCACTGTTGTCAC ‐3′

### 2.10 PCR quantitative analysis

Protein‐related PCR uses GAPDH as an internal reference, and microRNA‐related PCR uses U6 as an internal reference. Real‐time quantitative PCR was performed by the StepOnePlus Real‐Time PCR System. Use the PCR CT value of the target molecule minus the CT value of the internal control as the initial data ΔCT. The CT value of the control group is subtracted from the CT value of the experimental group, and then the negative value of the above result is −ΔΔCT as the initial value of calculation. Perform exponential processing of 2 for the above result, which is 2^−ΔΔCT^, and this result is the final statistical result. For clinical tissue specimens, we have a special processing method that uses log_2_(2^‐ΔCT^) as the final statistical data.

### 2.11 Western blot assays

Protease inhibitor cocktail and PMSF were added to RIPA protein lysis buffer (Wuhan Servicebio technology) for protein extraction. We took 40 μg of the above product for gel electrophoresis, and transferred the electrophoresis product to polyvinylidene fluoride membranes. Then, at room temperature, we used 5% milk to block membranes for 1 hour. Subsequently, primary antibodies were used to incubate the membrane above. Before detection, rinsed the membranes several times by PBST and incubated the membranes at room temperature for 2 hours by with secondary antibodies. The relevant antibodies information was shown as follows: NNT (Abclonal, A4561), PGC1A (Abclonal, A12348), HIF2a (Abclonal, A7553), UCP1 (Abcam, ab10983), GAPDH (Proteintech, 60004‐1‐Ig), DIO2 (Proteintech, 26513‐1‐AP), and CIDEA (Santa Cruz, sc‐8730‐R). The statistical analysis of all western blots involved in the research is shown in supporting information 2 and 3. The original blots for western blots are shown in supporting information 4‐14.

### 2.12 Cell viability assays

We used 96‐well plates to detect cell viability with 2000 cells per well. CCK8 (YEASEN, China) with a concentration of 10% was used to detect the cell viability. And 0, 24, 48, 72 and 96 hours play as the time point for the cell viability measurement upon treatments, respectively.

### 2.13 Colony formation assays

To conduct the colony formation assays, 6‐well plate was used as a container with 1000 cells per well. Fourteen days is a point in time to assess the ability of colony formation. After cell culture for 2 weeks, cells are fixed by methanol, and 0.05% crystal violet staining was used to visualize the surviving colonies (>50 cells/colony).

### 2.14 Wound healing assays

Cultivate the cells in a six‐well plate with a confluency of 80% and use serum‐free medium for pretreatment. Cell scratches were made with a 10‐uL pipette tip. Then, rinse the cells with PBS to remove cell residues, and use microscope to obtain relevant cell migration pictures with 0, 12, and 24 hours as the time point.

### 2.15 Transwell assays

In order to assess the migratory and invasion ability, serum‐free medium was used to pretreat the cells 24 hours before the start of the formal experiment. Then, add the above cells in the top chamber of transwell chamber. For the invasion assay, we needed to add Matrigel in a ratio of 1:8 on the basis of the above. After 1 day of incubation, methanol was used to fix cells for 10 minutes. Finally, crystal violet was used for staining to show the cell distribution. The result image was taken by using microscope. The number of cells used for migration analysis is 5 × 10^4^ cells, and the number of cells used for invasion analysis is 1 × 10^5^ cells.

### 2.16 Oil red staining

Culture cells in a 6‐well plate to a confluency of 30%. Prepare the oil red dye in the ratio of saturated oil red and ultrapure water at 2:3. Remove the cell culture medium and wash twice with PBS. Four percent paraformaldehyde is used to fix the cells, remove the paraformaldehyde and let it dry naturally after 10 minutes. Add the prepared oil red fuel, stain at room temperature for 30 minutes, then use PBS to remove the excess oil red fuel, dry it and observe with a microscope.

### 2.17 Triglyceride detection

Cells were cultured in a 6CM dish, and then a cell suspension was prepared. Centrifuge at 1000 rpm for 10 minutes, discard the supernatant and leave the cell pellet. Repeat the above steps twice with PBS, and reserve the cell pellet for later use. Use 200 uL of 2% TritonX‐100 to lyse the cells for 40 minutes. Then add 2.5 uL of the above product to 250 uL working solution, incubate at 37°C for 10 minutes, and take part of the sample for protein quantification. Finally, a microplate reader was used for detection at a wavelength of 510 nm. The calculation formula is shown below:

$$\begin{array}{ccc} & & Triglyceride\;content\;=\frac{\left(Sample\;OD\;-\;Blank\;OD\right)}{\left(\;Calibration\;OD-\;Blank\;OD\right)}\;\\ & & \;\times \;Standard\;concentration÷Protein\;concentration \end{array}$$

### 2.18 Luciferase plasmid construction

NNT‐related luciferase plasmids were obtained from RiboBio (RiboBio, Guangzhou, China). We used PCR to design the amplification primers based on the NNT (human) 3′ untranslated region (UTR) sequence information. Use PCR to amplify the 3′UTR sequence of the NNT gene using 293T genomic DNA as a template, and clone the relevant sequence into pmiR‐RB‐REPORT dual luciferase report vector.

Truncated plasmids of promoter regions of miR‐455‐5p were obtained from TIANYI HUIYUAN China. Construction vector is pGL3‐Basic. The internal reference control used pRL‐TK. The specific construction sequences are shown in Figure S7.

**Table:** **Table C**: NNT Luciferase plasmid construction

Vector name	Mutation site
H‐NNT‐WT	N/A
H‐NNT‐MUT‐1	GGCACAT (566‐572 bp) mutation to CCGTGTA
H‐NNT‐MUT‐2	GCACATA (2355‐2361 bp) mutation to CGTGTAT;
	GGCACAT (2649‐2655 bp) mutation to CCGTGTA;
	GCACATA (2902‐2908 bp) mutation to CGTGTAT

**Table:** **Table D**: NNT luciferase plasmid amplification primer

Primer	Sequence
H‐NNT‐f(XhoI)	GGCGCTCGAGTTGAATTAATCATATCAAATCAG
H‐NNT‐r(NotI)	AATGCGGCCGCTACATTTACTTATTTTTTAATGTAT
H‐NNT‐MUT1‐f	ATTTCAAGCCGTGTATTTCTCACTACTATTTTA
H‐NNT‐MUT1‐r	GTGAGAAATACACGGCTTGAAATATAGACTCTG
H‐NNT‐MUT2‐1‐f	GAAAATGTCGTGTATTACACCATGGAATACTAT
H‐NNT‐MUT2‐1‐r	ATGGTGTAATACACGACATTTTCTTAATCAAGT
H‐NNT‐MUT2‐2‐f	ACCAACATCCGTGTAGTATACATATGTAGCAAA
H‐NNT‐MUT2‐2‐r	ATGTATACTACACGGATGTTGGTGTGCTGCACC
H‐NNT‐MUT2‐3‐f	GAGGTAATCGTGTATTTAATTAGAAAGATTTTG
H‐NNT‐MUT2‐3‐r	CTAATTAAATACACGATTACCTCAATTAGTTAT

### 2.19 Luciferase reporter assay

Culture cells in 48‐well plates. Lipofectamine 2000 (Invitrogen) was used for transfecting the complimentary DNA. Luciferase activity was measured by Dual‐Luciferase Assay reagent (Promega, E1910). The specific experimental steps are as follows: Luciferase assay is carried out on the third day after cell plasmid transfection (cell fusion degree 90%); use one volume of 5xPLB and four volumes of ultrapure water to prepare lysate; wash the cells twice with PBS, then add 65uL lysis buffer, and lyse for 15 minutes at room temperature. After the above lysis is completed, start the detection. First, take 20 uL of the above product and mix 100 uL LARII, and use fluorescent microplate reader to measure the firefly fluorescence. Next, add 100 uL of 1xStop&Glo to the above mixture, and measure Renilla fluorescence with fluorescence microplate reader. Finally, with Renilla fluorescence as an internal reference, the ratio of the measured value of firefly fluorescence to Renilla fluorescence can reflect the activation degree of the reporter gene.

### 2.20 Chromatin immunoprecipitation assay

SimpleChIP Enzymatic Chromatin IP Kit (Agarose Beads) #9002 obtained from CST was used to carry out chromatin immunoprecipitation (CHIP) assay. The relevant primers designed for miR‐455‐5p promoter used for CHIP analysis were shown as follows:

**Table:** **Table E**: List of primers for CHIP

Primer	Sequence
**Control**	
Forward	5′‐ CCAGTCCTGATCTCCTGACC‐3′
Reverse	5′‐ AAGGGCAGCTTGAGTGACTC‐3′
**Site A**	
Forward	5′‐ CAGCATTTTGGGAGGCCAAG‐3′
Reverse	5′‐ TACCGGGTTCAAGCGATTCT‐3′
**Site B**	
Forward	5′‐ GATAGTGGTAGAAGCCCCGG‐3′
Reverse	5′‐ TCGGTAGCATCCTTCTCCAC‐3′
**Site C**	
Forward	5′‐ GCACCTACCAGCATCCCT‐3′
Reverse	5′‐ CAAAGGCACATACCCTCACG‐3′

### 2.21 In vivo tumor implantation

The experimental animals are divided into two groups, namely the experimental group and the control group, each with five experimental animals. The experimental animals were obtained from Vital River Laboratory Animal Technology Co. Ltd. Xenografts tumor models were obtained by subcutaneous injection of tumor cells in nude mice within a total of 2 × 10^6^ cells. Evaluation of tumor cell metastasis ability through tail vein injection. We measured tumor size every 4 days, and the time point of the last measurement was 47 days. After obtaining tissue specimens, immunohistochemical staining was carried out by the method described above. All animal experiments were carried out with the authorization of the Institutional Animal Use and Care Committee of Tongji Medical College, Huazhong University of Science and Technology. The license number of the ethical review for the study is S1892.

### 2.22 Bioinformatics analysis

The relevant gene sets for molecular screening came from the oncomine database (https://www.oncomine.org). Among them, Gene set ② was derived from the mitochondrial metabolism project in the "RCC differentially expressed genes in Higgins Renal"; Gene set ③ was derived from the mitochondrial metabolism project in the "RCC differentially expressed genes in Yusenko Renal"; Gene set ④ was derived from the mitochondrial metabolism project in the "RCC differentially expressed genes in Lenburg Renal." Clinically relevant information about ccRCC was obtained from TCGA‐KIRC Database (http://www.cbioportal.org/public-porta). Related pathway analysis was carried out through gene set enrichment analysis (GSEA). The online databases (Microcosm target, MicroRNA.org, MIRWALK) were used to screen the microRNA targeted NNT. The binding sites between miR‐455‐5p and NNT were predicted by Targetscan (https://www.targetscan.org).

### 2.23 Microscopic image processing

The microscope images were acquired by UopView for digital camera. Among them, transwell‐related pictures were acquired at 200x magnification, oil red staining‐related pictures were acquired at 400x magnification, IHC‐related pictures were acquired at 200x and 400x magnifications, and hematoxylin‐eosin staining (H&E) staining‐related pictures were acquired at 200x and 400x magnifications. The basic parameters of image capture are exposure target 120, exposure time 350 milliseconds, gain 100%, color temperature 6503, tint 1000, saturation 128, and gamma value 1.00. Use UopView for parameter correction before grouping of comparatively meaningful experiments to ensure that the experiment is comparable and consistent.

### 2.24 Statistical analysis

SPSS Statistics 22.0 (IBM SPSS, Chicago, IL) and Excel 2016 (Microsoft) were used for statistical analysis. The analysis between two groups used *t*‐test, and the analysis of more than two groups used ANOVA. Among them, independent‐samples *t*‐test is used to test whether two independent samples come from a group with the same mean; paired‐samples *t*‐test is used to test whether the means of two paired groups are significantly different. Receiver operator characteristic (ROC) curves were used to assess the value of clinical diagnosis. Cox regression was used to analyze survival‐related risk factors. Correlation analysis was carried out through Pearson correlation coefficient. *P* < .05 meant the data were statistically significant.

## 3 RESULTS

### 3.1 NNT was downregulated and predicted a poor prognosis in ccRCC

**FIGURE 1:**
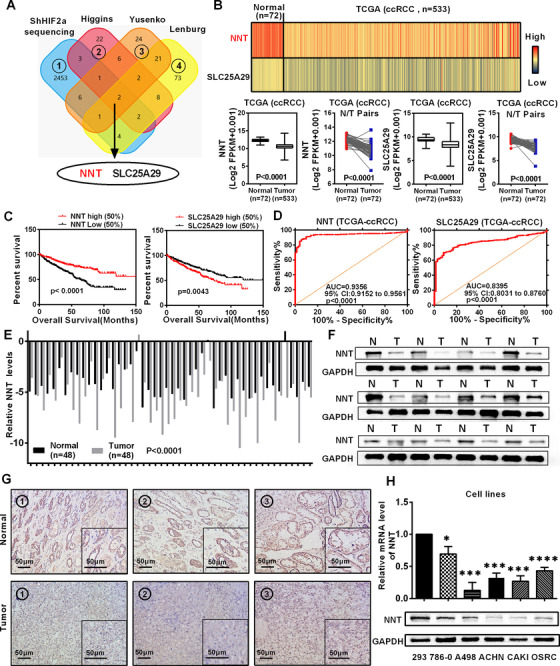
NNT was downregulated and predicted a poor prognosis in ccRCC. A, A venn diagram of three independent mitochondrial metabolism‐related gene sets from the oncomine database (https://www.oncomine.org) and the whole transcriptome sequencing data obtained after stable HIF2a knockdown. B, Levels of the NNT and SLC25A29 mRNAs in 533 ccRCC tissues and 72 paired tissues from patients with ccRCC based on data from the TCGA database. *t*‐test, *P* < .0001(independent‐samples *t*‐test for overall difference analysis, paired‐samples *t*‐test for N/T pairs analysis). C, The Kaplan‐Meier curves of NNT and SLC25A29 expression in patients with ccRCC for determining overall survival (OS) (log‐rank for statistics). D, The ROC (receiver operating characteristic) curves for NNT (AUC = 0.9356, 95% CI: 0.9152 to 0.9561; *P* < .0001) and SLC25A29 (AUC = 0.8395, 95% CI: 0.8031 to 0.8760; *P* < .0001) in ccRCC. E, Levels of the NNT mRNA in 48 ccRCC tissues and adjacent nonmalignant tissues (take the mRNA level of normal tissues as the control group). *t*‐test, *P* < .0001 (n = 48) (paired‐samples *t*‐test for statistics). F, Levels of the NNT protein in ccRCC tissues and adjacent nonmalignant tissues (n = 12). G, Immunohistochemical (IHC) staining for NNT in ccRCC tissues and adjacent nonmalignant tissues (scale bar: 50μm) (The tissues are derived from randomly selected tissue samples, where the same number is the paired tumor tissue and adjacent tissue of the same patient) (In the graph, the top three immunohistochemistry pictures represent three different normal tissue specimens, and the bottom three immunohistochemistry pictures represent three corresponding ccRCC tissue specimens. The illustration in the lower right corner of each immunohistochemistry picture is the partial magnified renderings of immunohistochemical pictures). H, Levels of the NNT mRNA and protein in five ccRCC cell lines and a normal cell line (n = 3). *t*‐test, *P < .05, ***P < .001, and ****P < .0001 (independent‐samples *t*‐test for statistics). Abbreviations: AUC, area under curve; ccRCC, clear cell renal cell carcinoma; CI, confidence interval; N, normal; NNT, nicotinamide nucleotide transhydrogenase; T, tumor; TCGA, The Cancer Genome Atlas

ccRCC is characterized by metabolic reprogramming, and the mitochondria are important organelles in the mediation of a variety of metabolic processes which are described as metabolic signaling centers within cells.^24^, ^25^ HIF2a is the most important oncogene in ccRCC, which is widely involved in the progression and metabolic dysfunction of ccRCC.^12^ To elucidate the mechanism of metabolic abnormalities in ccRCC, we screened sequencing data after HIF2a knockdown and three independent mitochondrial metabolism‐related gene set. Two of these genes showed differential expression in ccRCC (Figure 1A, Tables S1‐S3). We used the TCGA‐KIRC database to analyze the expression of these two genes in ccRCC. As shown in Figure 1B, both of these two genes showed significantly lower expression in ccRCC. Kaplan‐Meier curves based on TCGA database indicated that NNT expression was positively correlated with survival time, while a lower SLC25A29 level produced the opposite result, as the survival time was prolonged in the presence of a lower expression level (Figure 1C). ROC curves were used to evaluate clinical diagnostic value. The results also showed that NNT had higher clinical diagnostic value than SLC25A29, due to its larger AUC (Figure 1D). At the same time, the results of the whole transcriptome sequencing and TCGA analyses revealed a negative correlation between NNT and HIF2a, which is logically correct under the premise that HIF2a functions as an important oncogene for ccRCC (Figures S1A and S1B). After comprehensively considering these results, we focused on NNT in subsequent experiments.

The biological significance of NNT in ccRCCC was confirmed by further bioinformatics analysis. Imprimis, the six data sets derived from the Oncomine database further confirmed the low expression characteristics of NNT in ccRCC (Figure S1C). Then, more in‐depth analysis based on TCGA‐KIRC database showed that the low NNT expression also predicted a shorter disease‐free survival time, and it also had a discriminating ability between tumor and para‐tumor tissues (Figures S1D and S1E). At the same time, NNT expression showed a gradual decrease as the tumor stage and grade increased (Figure S1F). And in terms of clinicopathological parameters of ccRCC, it had a highly suggestive effect (Table 1). Moreover, subgroup analysis of the Kaplan‐Meier curves according to the data from TCGA‐KIRC database showed that the low NNT expression indicated a worse prognosis for both overall survival and disease‐free survival (Figures S2 and S3). Cox regression analysis according to TCGA‐KIRC database indicated that NNT was considered as an independent prognostic predictor in ccRCC (Tables 2 and 3).

**TABLE 1:** Correlation between NNT mRNA expression and clinicopathological parameters of ccRCC patients

			NNT mRNA expression		
Parameter		Number	Low (n = 258)	High (n = 259)	*P* value
Age (years)	< = 60	257	131	126	
	>60	260	127	133	.629
Gender	Male	336	173	163	
	Female	181	85	96	.326
T stage	T1 + T2	332	146	186	
	T3 + T4	185	112	73	.000^*^
N stage	N0 + NX	503	247	256	
	N1	14	11	3	.030^*^
M stage	M0 + MX	441	208	233	
	M1	76	50	26	.003^*^
G stage	G1 + G2	239	113	126	
	G3 + G4	278	145	133	.269
TNM stage	I + II	314	132	182	
	III + IV	203	126	77	.000^*^

^*^ means the result is statistically significant

The statistical method involved in the table is the chi‐square test.

**TABLE 2:** Univariate and multivariate analyses of NNT mRNA level and patient overall survival (OS)

	Univariate analysis			Multivariate analysis^c^		
Variable	HR^a^	95% CI^b^	*P* value	HR	95% CI	*P* value
Overall survival (n = 517)						
Age (years)						
≤60 (n = 257)	1.766	1.297‐2.404	.000^*^	1.661	1.219‐2.264	.001^*^
>60 (n = 260)						
Gender						
Female (n = 181)	0.965	0.707‐1.318	.825			
Male (n = 336)						
T stage						
T1 or T2 (n = 332)	3.043	2.245‐4.124	.000^*^	1.535	1.073‐2.195	.019^*^
T3 or T4 (n = 185)						
N stage						
N0 or NX (n = 503)	3.554	1.871‐6.748	.000^*^			
N1 (n = 14)						
M stage						
M0 or MX (n = 441)	4.369	3.197‐5.971	.000^*^	2.720	1.904‐3.885	.000^*^
M1 (n = 76)						
G grade						
G1 or G2 (n = 239)	2.605	1.853‐3.661	.000^*^	1.747	1.211‐2.520	.003^*^
G3 or G4 (n = 278)						
NNT						
Low (n = 258)	0.431	0.314‐0.593	.000^*^	0.535	0.386‐0.740	.000^*^
High (n = 259)						

^*^ means the result is statistically significant

^a^ Hazard ratio (HR), estimated from Cox proportional hazard regression model.

^b^ Confidence interval (CI) of the estimated HR.

^c^ Multivariate models are adjusted for T (tumor), N (lymph node), M (metastasis) classification, age, and gender.

**TABLE 3:** Univariate and multivariate analyses of NNT mRNA level and patient disease‐free survival (DFS)

	Univariate analysis			Multivariate analysis^c^		
Variable	HR^a^	95% CI^b^	*P* value	HR	95% CI	*P* value
Disease‐free survival (n = 421)						
Age (years)						
≤60 (n = 228)	1.363	0.957‐1.941	.086			
>60 (n = 193)						
Gender						
Female (n = 142)	1.421	0.956‐2.111	.082			
Male (n = 279)						
T stage						
T1 or T2 (n = 282)	4.503	3.117‐6.504	.000^*^	2.050	1.349‐3.117	.001^*^
T3 or T4 (n = 139)						
N stage						
N0 or NX (n = 409)	5.915	2.969‐11.781	.000^*^	2.563	1.256‐5.231	.010^*^
N1 (n = 12)						
M stage						
M0 or MX (n = 370)	8.494	5.852‐12.328	.000^*^	5.083	3.357‐7.695	.000^*^
M1 (n = 51)						
G grade						
G1 or G2 (n = 207)	3.352	2.220‐5.061	.000^*^	2.328	1.513‐3.582	.000^*^
G3 or G4 (n = 214)						
NNT						
Low (n = 210)	0.506	0.350‐0.730	.000^*^	0.580	0. 398‐0.846	.005^*^
High (n = 211)						

^*^ means the result is statistically significant

^a^ Hazard ratio (HR), estimated from Cox proportional hazard regression model.

^b^ Confidence interval (CI) of the estimated HR.

^c^ Multivariate models are adjusted for T (tumor), N (lymph node), M (metastasis) classification, age, and gender.

Subsequently, NNT protein and mRNA levels in ccRCC tissues were tested to verify the relevant results of bioinformatics analysis. As shown in Figures 1E‐1G, both NNT protein and mRNA levels were significantly lower in ccRCC. Furthermore, similar results were also obtained from ccRCC cell lines (Figure 1H).

### 3.2 NNT suppressed the progression of ccRCC

**FIGURE 2:**
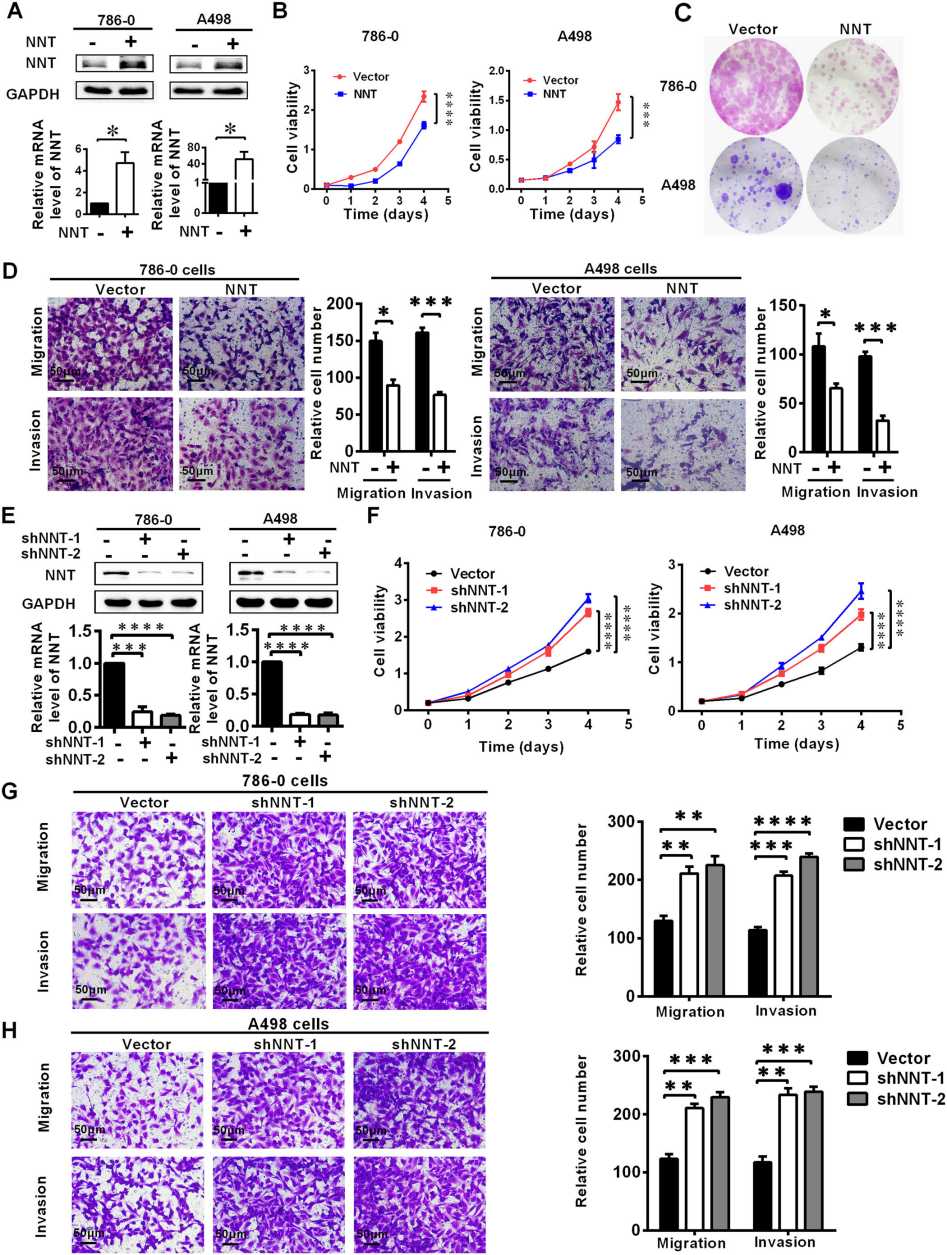
NNT suppressed the progression of ccRCC. NNT‐overexpressing or NNT knockdown ccRCC cell lines were constructed by transducting an overexpressing lentivirus and shRNA, respectively. The results are plotted as the means ± SEM from three independent experiments with at least three replicates in each independent experiment. ****P < .0001, ***P < .001, **P < 0.01, and *P < .05. A, The overexpression of NNT was verified at the protein and mRNA levels using western blotting and qPCR, respectively (n = 3) (independent‐samples *t*‐test for statistics). B, Cell growth curves of CCK8 assays for the cell lines overexpressing NNT (n = 4) (independent‐samples *t*‐test for statistics). C, The result of colony formation assay for the cells overexpressing NNT. D, The results of transwell assay of the migration and invasion for the cells overexpressing NNT (n = 3, scale bar: 50μm) (independent‐samples *t*‐test for statistics). E, The NNT levels in the cells with NNT knockdown were verified by western blotting and qPCR (n = 3) (independent‐samples *t*‐test for statistics). F, Cell growth curves of CCK8 assays for the cell lines with NNT knockdown (n = 4) (independent‐samples *t*‐test for statistics). G and H, The results of transwell assays of migration and invasion for the cells with NNT knockdown (n = 3, scale bar: 50μm) (independent‐samples *t*‐test for statistics)

The significant expression difference of NNT in ccRCC suggested that it was likely to have a potential effect on the biological function of ccRCC. In order to clarify the effect of NNT on the biological function of ccRCC, we used expression lentivirus and shRNA to construct cell lines with NNT stably overexpression and stably knockdown in A498 and 786‐0 cell lines (Figures 2A and 2E). The results of CCK8 cell viability analysis showed that cell lines with NNT overexpression significantly reduced the proliferation ability of ccRCC (Figure 2B). Conversely, NNT knockdown cell lines tended to exhibit increased proliferation (Figure 2F). The colony formation assay suggested the similar results (Figure 2C). The results of transwell and wound healing assays showed that the migration and invasion ability of cell lines with NNT overexpression were significantly reduced (Figure 2D, Figure S4), while the migration and invasion were improved following NNT knockdown (Figures 2G and 2H). These results demonstrate that NNT acts as a tumor suppressor to inhibit the progression of ccRCC.

### 3.3 NNT functioned as a key molecule to affect lipid accumulation in ccRCC

**FIGURE 3:**
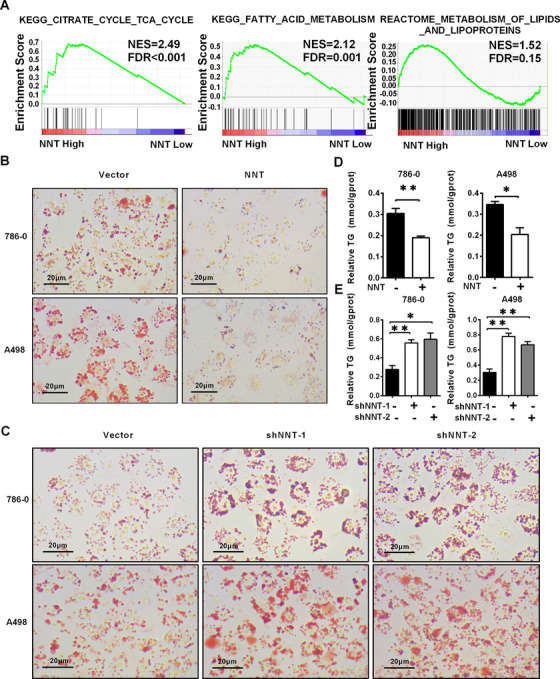
NNT functioned as a key molecule to affect lipid accumulation in ccRCC. A, GSEA (gene set enrichment analysis) assays for the correlations between the TCA cycle, fatty acid metabolism and lipid/lipoprotein metabolism in ccRCC with the levels of the NNT mRNA, according to the TCGA database. FDR < 25% and *P* < .05 were considered statistically significant. B, Photomicrographs of Oil Red O staining of the cell lines overexpressing NNT compared with the negative control (n = 3, scale bar: 20μm). C, Photomicrographs of Oil Red O staining in NNT knockdown cells (n = 3, scale bar: 20μm). D and E, Relative TG (mmol/gprot) levels in cells with NNT overexpression and knockdown assessed by a triglyceride assay kit (n = 3). *P < .05 and **P < .01 (independent‐samples *t*‐test for statistics). Abbreviations: FDR, false discovery rate; KEGG, Kyoto Encyclopedia of Genes and Genomes; NES, normalized enrichment score; TCA, tricarboxylic acid cycle; TG, Triglycerides

Notably, dysfunctional lipid metabolism is an important feature of ccRCC.^26^ Abnormal lipid accumulation plays a vital role in ccRCC progression, and it is an important mediator of the HIF2a‐induced carcinogenic process.^12^ By analyzing the TCGA‐KIRC database through the GSEA, we found that NNT was involved in the TCA cycle, fatty acid metabolism, and lipid/lipoprotein metabolism in ccRCC (Figure 3A). Therefore, NNT is very likely to exert a potential effect on lipid metabolism in ccRCC. To test this hypothesis, the results of oil red staining showed that the lipid accumulation was obviously decreased in ccRCC cell lines overexpressing NNT, while a significant increase in lipid accumulation was observed in the NNT knockdown cell lines (Figures 3B and 3C). Subsequently, triglyceride contents were measured as a quantitative indicator of lipid accumulation in ccRCC. By using the triglyceride assay kit, we measured the triglyceride contents in the ccRCC cell lines in which NNT was overexpressed and knocked down. Similar results to oil red staining were obtained in which cell lines overexpressing NNT exhibited a relatively lower triglyceride content, while a relatively higher triglyceride content was detected in the NNT knockdown cells (Figures 3D and 3E). Overall, NNT is regarded as a key factor that regulates lipid accumulation in ccRCC.

### 3.4 NNT alleviated lipid accumulation by activating lipid browning‐mediated tumor cell “slimming” in ccRCC

**FIGURE 4:**
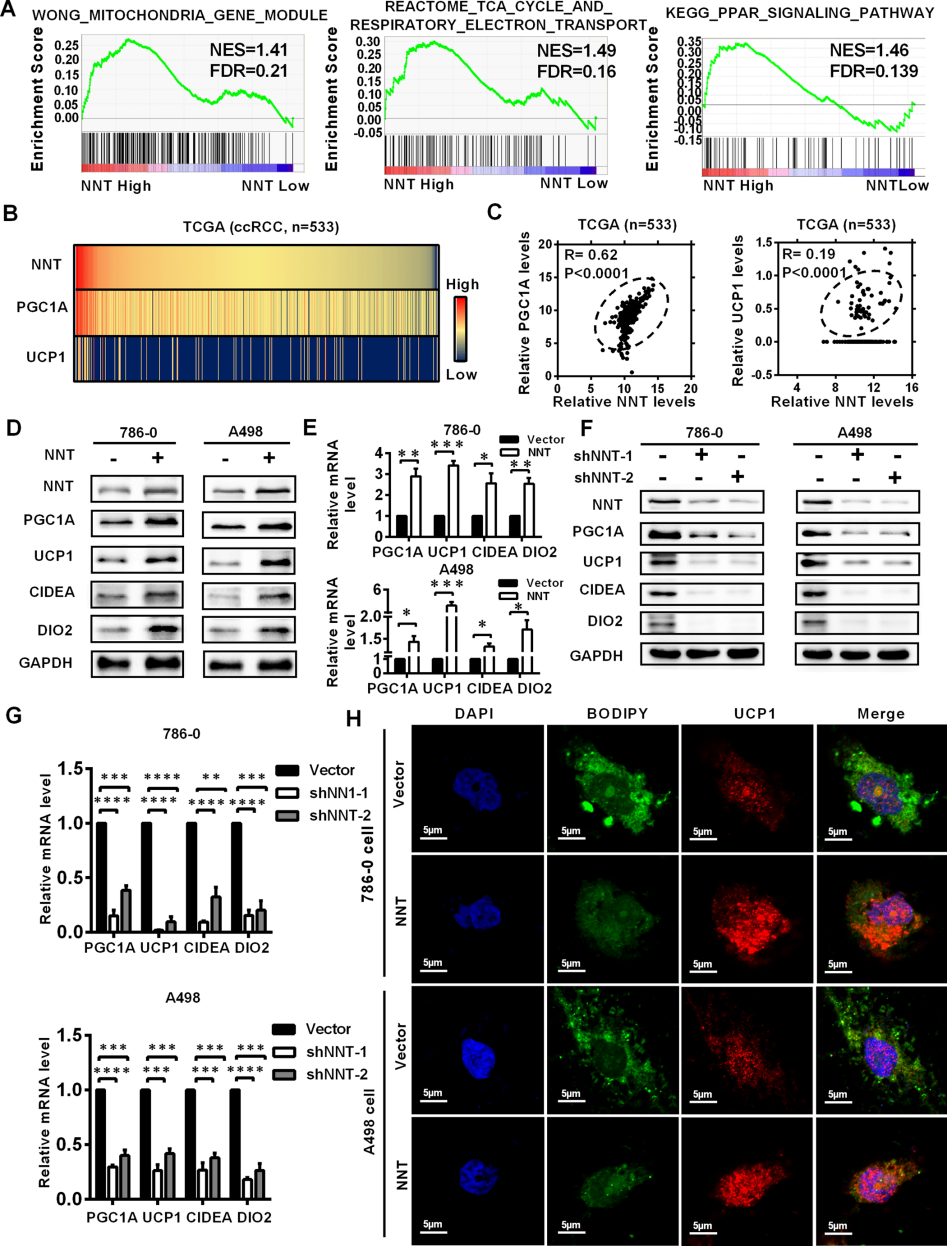
NNT alleviated lipid accumulation by activating lipid browning‐mediated tumor cell “slimming” in ccRCC. A, GSEA (gene set enrichment analysis) assays for the correlations between the mitochondria, respiratory electron transport chain, and PPAR signaling pathway in ccRCC with the levels of the NNT mRNA, according to the TCGA database. FDR < 25% and *P* < .05 were considered statistically significant. B and C, The correlation heatmap and the linear correlation curve between NNT and the most critical molecules related to lipid browning (PGC1A and UCP1) based on the data from the TCGA‐KIRC database (Pearson correlation coefficient for statistics). D‐G, Protein and mRNA levels of lipid browning marker genes (PGC1A, UCP1, CIDEA, and DIO2) in the cell lines with NNT overexpressed and knocked down assessed by western blotting and qPCR, respectively (n = 3) (independent‐samples *t*‐test for statistics). H, Representative photographs of immunofluorescence staining for lipids with BODIPY 493 of 503 (green) and lipid browning with UCP1 (red) in the cells overexpressing NNT (scale bar: 5μm). Abbreviations: ccRCC, clear cell renal cell carcinoma; DAPI, 4′,6‐diamidino‐2‐phenylindole; FDR, false discovery rate; KEGG, Kyoto Encyclopedia of Genes and Genomes; NES, normalized enrichment score; PPAR, peroxisome proliferators‐activated receptor; TCA, tricarboxylic acid cycle; TCGA, The Cancer Genome Atlas

To clarify the mechanism by which NNT regulated lipid metabolism, we conducted further analysis. As shown in Figure 4A, the results of GSEA indicated that the function of NNT was highly enriched in mitochondria, respiratory electron transport, and the peroxisome proliferators‐activated receptor (PPAR) signaling pathway. Tumor cell “slimming” is a newly defined concept which can efficiently consume tumor lipids and significantly inhibit tumor progression without producing ATP.^16^ Since the tumor cell “slimming” is mediated by lipid browning‐like reaction in tumors which is highly correlated with the PPAR signaling pathway, and it occurs exactly in mitochondria, there is reason to believe that NNT has a strong potential link with tumor cell “slimming.”

To confirm this hypothesis, according to the data from the TCGA‐KIRC database, we plotted the correlation heatmap and correlation linear curve between the most important marker genes of lipid browning (PGC1A and UCP1) and NNT. The results indicated that NNT had a significant positive correlation with the key marker genes of lipid browning (Figures 4B and 4C). Meanwhile, based on the oil red staining shown in Figure 3B, cell lines overexpressing NNT consumed lipids accompanied with LDs transformed into tiny pieces, and this phenomenon is exactly the hallmark features of lipid browning‐mediated tumor cell “slimming.”^16^ Similar conclusions were drawn from the NNT knockdown cells (Figure 3C). Then, western blotting and qPCR were performed to clarify the correlation between NNT levels and lipid browning. Significantly higher mRNA and protein levels of the PGC1A, UCP1 and other markers of lipid browning (CIDEA and DIO2) were detected in the ccRCC cell lines overexpressing NNT,^27^ while their levels were obviously decreased in the NNT knockdown cells (Figures 4D‐4G). Subsequently, we used BODIPY 493 of 503 and the most important molecule of lipid browning (UCP1) to identify the LDs and lipid browning level in the cells overexpressing NNT through immunofluorescence staining. The results also showed that the decrease in lipid accumulation observed in the NNT‐overexpressing cells was accompanied by an increase in the level of lipid browning (Figure 4H). Therefore, we concluded that NNT achieves lipid consumption in ccRCC by inducing lipid browning‐mediated tumor cell “slimming.”

### 3.5 NNT‐mediated tumor cell “slimming” reversed the pro‐carcinogenesis effect of HIF2a in ccRCC

**FIGURE 5:**
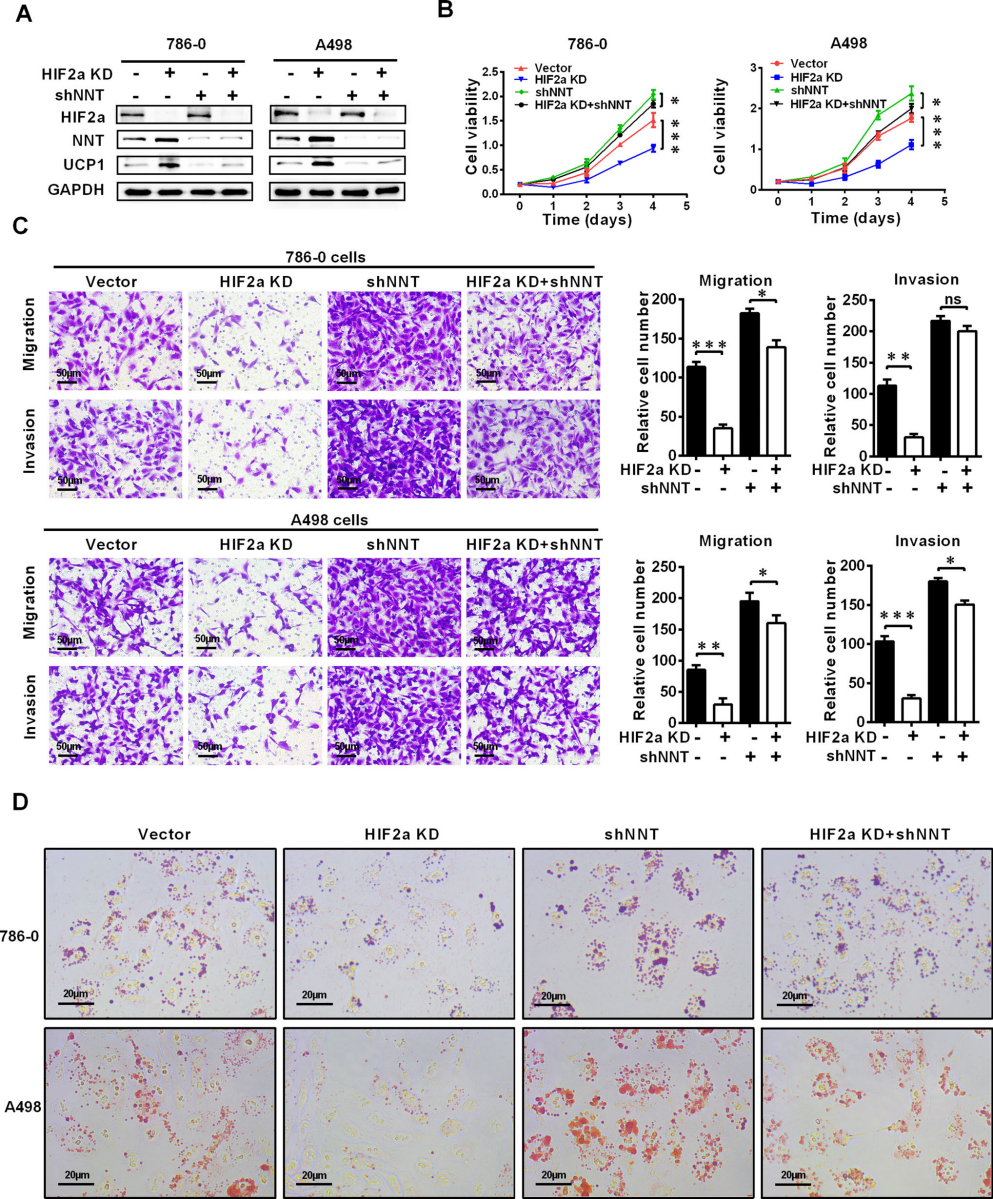
NNT‐mediated lipid browning reversed the pro‐carcinogenesis effect of HIF2a on ccRCC. The ccRCC cell lines in which NNT was knocked down were constructed by transfecting shRNA into cells with stable HIF2a knockdown (HIF2a KD stands for HIF2a knockdown, which is constructed by cell lines transduction with shHIF2a lentivirus). A, Western blot showing levels of the HIF2a, NNT, and UCP1 proteins in the indicated cell lines (n = 3). B, Cell growth curves of CCK8 assays for indicated cell lines (n = 4). ****P < .0001, ***P < .001, and **P < .01 (independent‐samples *t*‐test for statistics). C, Transwell assays of migration and invasion were conducted for the indicated ccRCC cell lines (n = 3, scale bar: 50μm). *t*‐test, ***P < .001, **P < .01, *P < .05, *P* = ns (not significant) (independent‐samples *t*‐test for statistics). D, Photomicrographs of Oil Red O staining for the cells described above (n = 3, scale bar: 20μm)

Since NNT was identified in sequencing results of ccRCC cell line with HIF2a knockdown, a close correlation between the functions of NNT‐mediated pathway and HIF2a likely exists. Functional rescue experiments were conducted to further illustrate the importance of NNT‐mediated pathway in regulating HIF2a function in ccRCC. Due to the negative regulatory relationship between HIF2a and NNT (Figures S1A and S1B), we constructed ccRCC cell lines with NNT knockdown by shRNA based on stable HIF2a knockdown (Figure 5A). Previous studies have confirmed that HIF2a functions as an oncogene to promote the progression of ccRCC. In this study, the results of CCK8 cell viability analysis also showed that knockdown of HIF2a significantly inhibited cell proliferation. Moreover, NNT silencing significantly alleviated the inhibition of ccRCC proliferation caused by HIF2a knockdown (Figure 5B). Studies on invasion and migration could draw similar conclusions. As shown in Figure 5C, HIF2a knockdown significantly inhibited the migration and invasion ability of ccRCC cell lines, and further silencing of NNT partially reversed this biological effect. Then, oil red staining was also performed to show changes in lipid metabolism in ccRCC cells during this process. It is obvious that NNT silencing largely reversed the effect of lipid metabolism caused by HIF2a silencing in ccRCC cells (Figure 5D). The level of lipid browning described above was also tested by assessing the expression of its most important marker, UCP1. Again, the results were similar, as NNT silencing largely reversed the biological effects of HIF2a silencing (Figure 5A). In summary, NNT‐mediated tumor cell “slimming” can reverse the carcinogenic effect of HIF2a in ccRCC, which can be considered as an important downstream factor of HIF2a.

### 3.6 HIF2a regulated NNT expression via miR‐455‐5p

**FIGURE 6:**
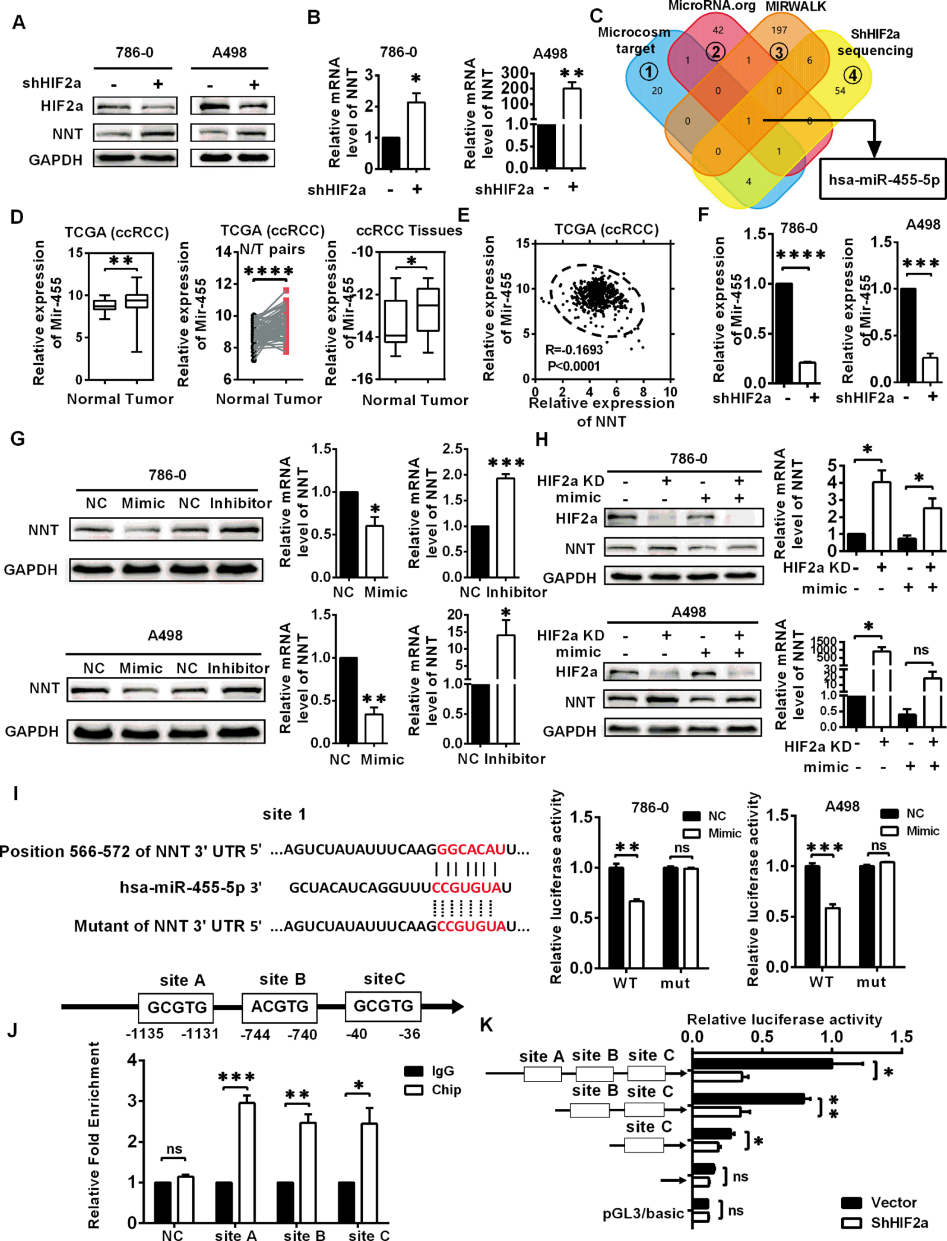
HIF2a regulated NNT expression via miR‐455‐5p. In the graph, HIF2a KD stands for HIF2a knockdown, which is constructed by cell lines transduction with shHIF2a lentivirus. A and B, Levels of the NNT protein and mRNA in the HIF2a knockdown cells (n = 3) (independent‐samples *t*‐test for statistics). C, The microRNAs predicted to target NNT by online databases (Microcosm target, MicroRNA.org, MIRWALK) and microRNAs that were downregulated in the HIF2a knockdown cells according to the sequencing data are shown. D, Levels of miR‐455‐5p in TCGA‐KIRC database and ccRCC samples. *t*‐test, *P < .05, **P < .01, and ****P < .0001 (independent‐samples *t*‐test for overall difference analysis, paired‐samples *t*‐test for N/T pairs analysis). E, Clinical correlations of NNT expression with miR‐455‐5p expression in ccRCC tumors were analyzed by linear regression based on the data from the TCGA database (Pearson correlation coefficient for statistics). F, Levels of miR‐455‐5p in the HIF2a knockdown cells (n = 3) (independent‐samples *t*‐test for statistics). G, Protein and mRNA levels of NNT in 786‐0 and A498 cells in which miR‐455‐5p was overexpressed or silenced after treatment with miR‐455‐5p mimic or inhibitor, respectively (mimic refers to miRNA mimics, which are double‐stranded sequences that are artificially synthesized to simulate endogenous miRNAs. Inhibitor refers to miRNA inhibitor, which is a single‐stranded sequence that uses artificial chemical synthesis to specifically inhibit target miRNA) (n = 3) (independent‐samples *t*‐test for statistics). H, Protein and mRNA levels of NNT in 786‐0 and A498 cells with/without knockdown of HIF2a and overexpression of miR‐455‐5p (n = 3) (independent‐samples *t*‐test for statistics). I, The base sequence on the left represents the binding sequence between miR‐455‐5p and NNT and the construction of the luciferase reporter plasmid. The bar chart on the right represents the results of luciferase reporter assays between miR‐455‐5p and NNT (n = 3) (independent‐samples *t*‐test for statistics). J, Potential HIF2a binding site in miR‐455‐5p promoter and the result of CHIP assay between miR‐455‐5p and HIF2a (n = 3) (independent‐samples *t*‐test for statistics). K, Promoter truncation showing that HIF2a binding to region site B (−744 ‐ −740) of miR‐455‐5p promoter is important for HIF2a's regulation of miR‐455‐5p (n = 3) (independent‐samples *t*‐test for statistics) Abbreviations: ccRCC, clear cell renal cell carcinoma; CHIP, chromatin immunoprecipitation assay and promoter analysis; mut, mutant; N, normal; NC, negative control; ns, not significant; T, tumor; TCGA, The Cancer Genome Atlas; UTR, untranslated region; has, homo sapiens; WT, wild type

As a transcription factor HIF2a generally promotes the expression of downstream target genes,^28^, ^29^ while there is a negative regulation between NNT and HIF2a (Figures 6A and 6B, Figure S5A), we have introduced microRNAs as a bridge to explain the negative regulatory relationship between HIF2a and NNT. MicroRNAs (miRNAs) are a type of small noncoding RNAs with the function of inhibiting gene expression, which target the 3′ UTR of the mRNAs to block translation or degrade target mRNAs.^30^ Considering the negative relationship between NNT and HIF2a and the negative regulation of microRNA and target genes, HIF2a should logically exhibit a positive regulatory relationship with potential microRNAs. By screening microRNAs which target NNT using online databases (Microcosm target, MicroRNA.org, and MIRWALK) and identifying microRNAs that were downregulated in the HIF2a knockdown cells according to the sequencing data, miR‐455‐5p was the only microRNA identified which met these criteria (Figure 6C). Based on the verification of the TCGA‐KIRC database and our own tissue samples, miR‐455‐5p was expressed at high levels in ccRCC, consistent with the logic of our hypothesis (Figure 6D). In addition, analysis according to the TCGA‐KIRC database also indicated that HIF2a and miR‐455 are positively correlated, and miR‐455 and NNT are negatively correlated (Figure 6E, Figure S5B). Subsequently, the positive regulation between HIF2a and miR‐455‐5p was confirmed by qPCR in ccRCC cell lines (Figure 6F).

To verify the regulation between NNT and miR‐455‐5p, specific mimic and inhibitor were used to change the miR‐455‐5p expression (Figures S5C and S5D). As shown in Figure 6G, high miR‐455‐5p levels were accompanied with low levels of NNT, while low miR‐455‐5p levels were accompanied with high levels of NNT. To further clarify the relationship between NNT, HIF2a, and miR‐455‐5p, an interruption approach was conducted. As expected, overexpression of miR‐455‐5p partially reversed the increase in NNT expression caused by HIF2a knockdown at both the protein and mRNA levels (Figure 6H). Therefore, HIF2a might function by inducing miR‐455‐5p expression to regulate NNT expression.

Luciferase reporter assays were performed to confirm the specific mechanism by which miR‐455‐5p regulated NNT expression. Based on TargetScan prediction, one relatively strong and three relatively weak binding sites for miR‐455‐5p are located in the 3′ UTR of NNT, named sites 1‐4. The results showed that overexpression of miR‐455‐5p obviously inhibited the luciferase activity of wild‐type NNT 3′‐UTR and NNT 3′‐UTR with mutations at sites 2, 3, and 4 while statistically significant suppression of luciferase reporter activity was not observed for the NNT 3′‐UTR mutated at site 1, suggesting that miR‐455‐5p might target NNT through the 3′‐UTR site 1 (Figure 6I, Figures S5E and S5F).

Subsequently, we explored the specific mechanism by which HIF2a regulates miR‐455‐5p. According to HIF2a binding sequence,^31^ we found three potential HIF2a binding sites named sites A, B, and C in the 2000 bp region upstream of the miR‐455‐5p transcriptional start site (Figure S6A). As shown in Figure 6J, the results of CHIP assay indicated that all predicted sites of miR‐455‐5p could bind to HIF2a in the ccRCC cell line. Moreover, luciferase reporter assays conducted by truncated plasmids of this region further confirmed that HIF2a might mainly function by binding to site B, and to a lesser extent by binding to sites A and C (Figure 6K, Figure S7A). These results indicate that HIF2a increases miR‐455‐5p expression via binding to HIF2a‐related responsive elements in the miR‐455‐5p promoter region, which subsequently suppresses NNT expression by binding to its 3′ UTR.

### 3.7 NNT‐mediated tumor cell “slimming” suppressed ccRCC progression in vivo

**FIGURE 7:**
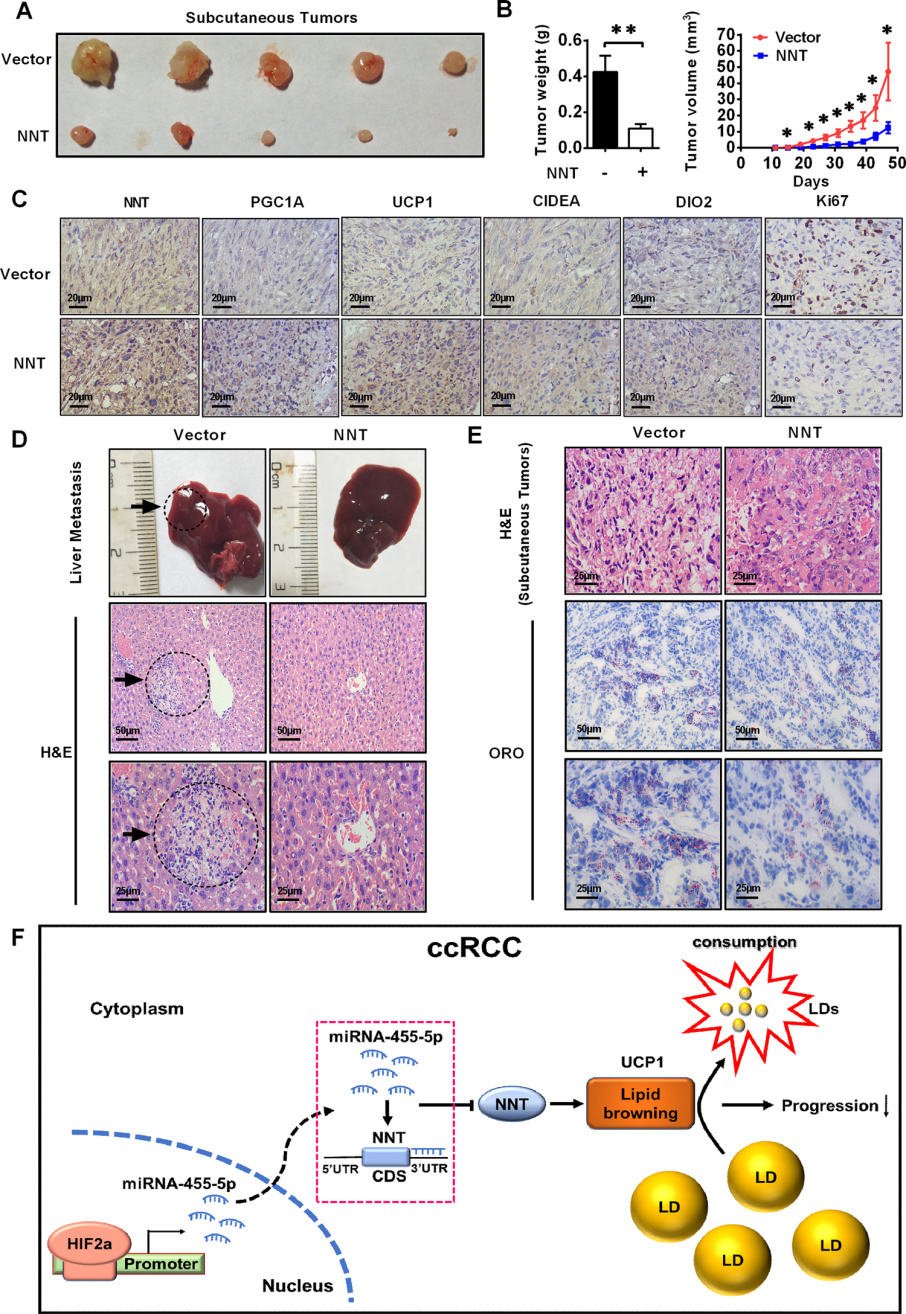
NNT‐mediated pathway suppressed ccRCC progression in vivo. A and B, A498 cells stably overexpressing NNT were injected into nude mice. The tumor size was measured every 4 days. The data are presented as the means ± SEM from separate tumors of each group. Images of tumors dissected from the mice. The tumor size (mm^3^) was plotted against days after tumor cell implantation. Tumors were weighted after resection at the end of the experiment (n = 5) (independent‐samples *t*‐test for statistics) (Tumor size was measured every 4 days, and the last measurement was performed on day 47). C, Immunohistochemical (IHC) staining for markers of lipid browning (PGC1A, UCP1, CIDEA, and DIO2) and the marker of tumor malignancy (KI67) in the tumor xenografts (Scale bar: 20μm). D, H&E staining of the liver tissues from the NNT‐overexpressing and control groups (scale bar: 50μm and 25μm). E, H&E staining and Oil Red O staining of the tumor xenografts from the NNT‐overexpressing and control groups (scale bar: 50μm and 25μm). F, A model could be suggested in which HIF2a increases miR‐455‐5p expression by binding to HIF2a response elements in the miR‐455‐5p promoter, which suppresses NNT expression by binding to its 3′ UTR. Meanwhile, NNT promotes lipid browning to consume lipids, resulting in the suppression of ccRCC progression. Abbreviations: ccRCC, clear cell renal cell carcinoma; CDS, coding sequence; H&E, hematoxylin‐eosin staining; LD, lipid droplet; ORO, Oil Red O; UTR, untranslated region

After obtaining the above results, we tried to study the function of NNT at the level of animal models. We used subcutaneous injection to construct xenograft tumor models in nude mice using the A498 cell line with NNT overexpression. We measured tumor size every 4 days, and the time point of the last measurement was 47 days. The results showed that both the volume and weight of subcutaneous xenografts were significantly reduced in the group overexpressing NNT (Figures 7A and 7B). Based on the observation that NNT promoted lipid browning in ccRCC described above, IHC was used to evaluate the level of lipid browning and the degree of malignancy of xenografts tumors. As shown in Figure 7C, the levels of markers for lipid browning were significantly increased in the group overexpressing NNT, and KI67, the marker of tumor malignancy, was significantly decreased in NNT overexpression group. Thus, consistent with research results at the cellular level, NNT overexpression significantly reduced the malignancy of tumors and promoted lipid browning in vivo. The results of tail vein metastasis models showed that NNT overexpression significantly reduced the liver metastasis of tumor cells (Figure 7D). Subsequently, oil red and H&E staining were used as visual indicators of lipid accumulation in subcutaneous tumors. A decrease in lipid accumulation was observed in subcutaneous xenografts overexpressing NNT (Figure 7E).

Based on these findings, we created a new model in which HIF2a increases miR‐455‐5p expression via binding to HIF2a‐related responsive elements in the miR‐455‐5p promoter region, which suppresses NNT expression by binding to its 3′ UTR. Moreover, NNT promotes lipid browning to active tumor cell “slimming,” resulting in the suppression of ccRCC progression (Figure 7F).

## 4 DISCUSSION

The extensive activation of HIF2a and the abundance of intracellular LDs are two vital features of ccRCC. HIF2a is a key oncogene in ccRCC, and abnormal lipid accumulation is also a key factor in maintaining and promoting ccRCC progression.^12^ However, the specific mechanism of HIF2a regulation of lipid metabolism in ccRCC was not completely understood. Tumor cell “slimming” is an emerging concept in which cells activate lipid browning to efficiently consume lipids and inhibit tumor progression. The present study identified a new pathway of HIF2a regulation of lipid metabolism via NNT‐mediated lipid browning. Mechanistic investigations showed that HIF2a suppressed NNT expression by activating miR‐455‐5p, which reduced the level of lipid browning‐mediated tumor cell “slimming” and resulted in ccRCC progression.

HIF2a is considered to be a transcription factor, and its connection with lipid metabolism is rarely mentioned. Our current research showed the new mechanism of HIF2a regulated lipid browning. Lipid browning is a special process that utilizes the characteristics of UCP1 to promote lipid consumption without ATP production.^32^ As well‐recognized, abundant lipid accumulation occurs in ccRCC is similar to the occurrence of obesity, which is caused by the synthesis rate of lipids being greater than the decomposition rate.^26^, ^33^, ^34^ And obesity has become a potentially important risk factor for ccRCC.^35^, ^36^ Therefore, an inextricable relationship must exist between ccRCC and obesity. Lipid browning is the focus of research on lipid consumption in obese subjects.^37^ It is not simply a pathway of lipid consumption, but rather an ability to consume lipids. An increase in the lipid browning level significantly increases the ability of the body to consume lipids in response to the same stimulus.^38^, ^39^ Its main feature is the generation of heat without the production of ATP to disrupt energy homeostasis, which leads to increased energy expenditure, and then a positive feedback to promote the consumption of lipids is activated.^40^, ^41^ Current researchers attempted to use lipid browning as a potential target for cancer treatment, and we found that lipid browning caused tumor cell “slimming” in tumors resulting in the inhibition of tumor progression.^42^, ^43^ We believe that this process is equally applicable to ccRCC, as NNT can promote a process similar to lipid browning to set a new balance point of lipid metabolism in the direction of lipid consumption. Moreover, based on the observation that a large amount of ATP is required for tumor progression,^44^ the feature of this process in which heat is generated without ATP production allows ccRCC cells to consume lipids without extra energy production. This is more conducive to the inhibition of tumor progression. In the normal process of ccRCC, HIF2a suppressed the expression of NNT, thereby inhibiting lipid browning induced by NNT. In summary, this inhibitory process is likely an important mechanism for HIF2a promoting ccRCC progression and lipid accumulation.

HIF2a is the most critical oncogene in ccRCC, and it was extensively investigated in the research and treatment of ccRCC. Current studies of HIF2a primarily focus on its function in angiogenesis. The currently used first‐line drug in the treatment of ccRCC, sunitinib, is a class of drugs targeting the family of vascular endothelial growth factor receptors and platelet‐derived growth factor receptors, which are important downstream targets of HIF2a.^45^, ^46^, ^47^ Although sunitinib significantly improves the treatment of ccRCC, drug resistance remains a very common and inevitable problem.^48^ The disease usually develops after 6‐15 months, leading to a poor prognosis.^49^, ^50^ Recent studies demonstrated that the carcinogenic effect of HIF2a in ccRCC is not only related to angiogenesis, but also lipid metabolism is a nonnegligible factor. However, few reports assessed the treatment of lipid metabolism. The present study confirmed that NNT was an important molecule that regulated lipid metabolism via HIF2a in ccRCC. These results suggest that a treatment targeting the NNT‐mediated pathway would play a vital role in lipid metabolism in ccRCC. Our results support a new drug combination strategy using anti‐angiogenesis therapy and the activation of NNT using small molecule agonists targeting the NNT‐mediated pathway.

In addition, it is worth noting that tumor slimming mediated by lipid browning‐like reaction contains multiple functional links. As shown in our research, NNT can activate many key molecules in lipid browning. Combined with the comprehensive phenotype of the interaction between HIF2a and NNT, NNT plays a dominant role in the tumor slimming process mediated by HIF2a. At the same time, since UCP1 is the most direct effector molecule in lipid browning and is generally considered to be highly co‐expressed with genes related to lipid browning,^51^, ^52^, ^53^ therefore, we believe that other lipid browning‐related genes are also dependent on NNT in the process of HIF2a regulating tumor slimming. We also found that in the functional rescue experiment, the rescue of HIF2a on lipid metabolism by NNT was incomplete. Then, whether there is a bypass or other mechanism for HIF2a to directly regulate lipid browning genes has become the focus of our next stage of exploration. By discovering the bypass, perfecting the mechanism of HIF2a and lipid metabolism regulation will help the development of new drugs and improve the overall treatment efficiency.

## 5 CONCLUSION

The present study revealed a specific mechanism by which HIF2a decreases NNT levels via a microRNA that suppresses tumor cell “slimming,” resulting in the progression of ccRCC. Therefore, this study provides a novel understanding of HIF2a in the regulation of the progression ccRCC. These findings provide an opportunity for the development of new drugs and combined therapeutic strategies to improve the treatment status of patients with ccRCC and other tumors with lipid accumulation.

## 6 STUDY HIGHLIGHTS

The findings of this study clarify the mechanism by which HIF2a regulates lipids in ccRCC. At the same time, it is the first time to clarify the regulatory relationship between the most important carcinogen HIF2a in ccRCC and the new concept tumor “slimming,” in which HIF2a decreases the NNT level through miR‐455‐5 p that suppresses tumor cell “slimming,” resulting in the progression of ccRCC. And the study clarifies the status of NNT as a bridge between HIF2a and tumor “slimming.” These findings provide new directions and targets for the development of new drugs for tumor “slimming” and HIF2a. It might provide an opportunity for us to develop new drugs and combinated therapeutic strategies to improve the treatment status of patients with ccRCC, as well as other tumors with lipid accumulation.

## CONFLICT OF INTEREST

The authors declare that there is no conflict of interest that could be perceived as prejudicing the impartiality of the research reported.

## AUTHOR CONTRIBUTIONS

Xiaoping Zhang, Ke Chen, Zhiyong Xiong, and Hongmei Yang conceived the idea. Zhiyong Xiong, Wei Xiong, and Wen Xiao conceived and designed the experiments. Zhiyong Xiong, Wei Xiong (Wei Xiong), Wen Xiao, Changfei Yuan, and Jian Shi performed the experiments. Yu Huang and Cheng Wang helped with animal experiments. Xiangui Meng and Zhixian Chen helped to obtain clinical samples. Zhiyong Xiong, Wen Xiao, and Hongmei Yang analyzed the data. Zhiyong Xiong, Wei Xiong, and Ke Chen wrote the manuscript. All authors reviewed and approved the manuscript.

## Supporting information

Supporting Information

Supporting Information

## Data Availability

The data that supports the findings of this study are available in the supplementary materials and supporting information of this article.
