# Supplementary material for: NNT‐induced tumor cell “slimming” reverses the pro‐carcinogenesis effect of HIF2a in tumors
Source: Clin Transl Med. 2021 Jan 12;11(1):e264. doi: 10.1002/ctm2.264 (PMC7803359; doi:10.1002/ctm2.264)
Supplement: Supplementary file 1 — Supporting Information [file CTM2-11-e264-s001.pdf]

Supporting information 1

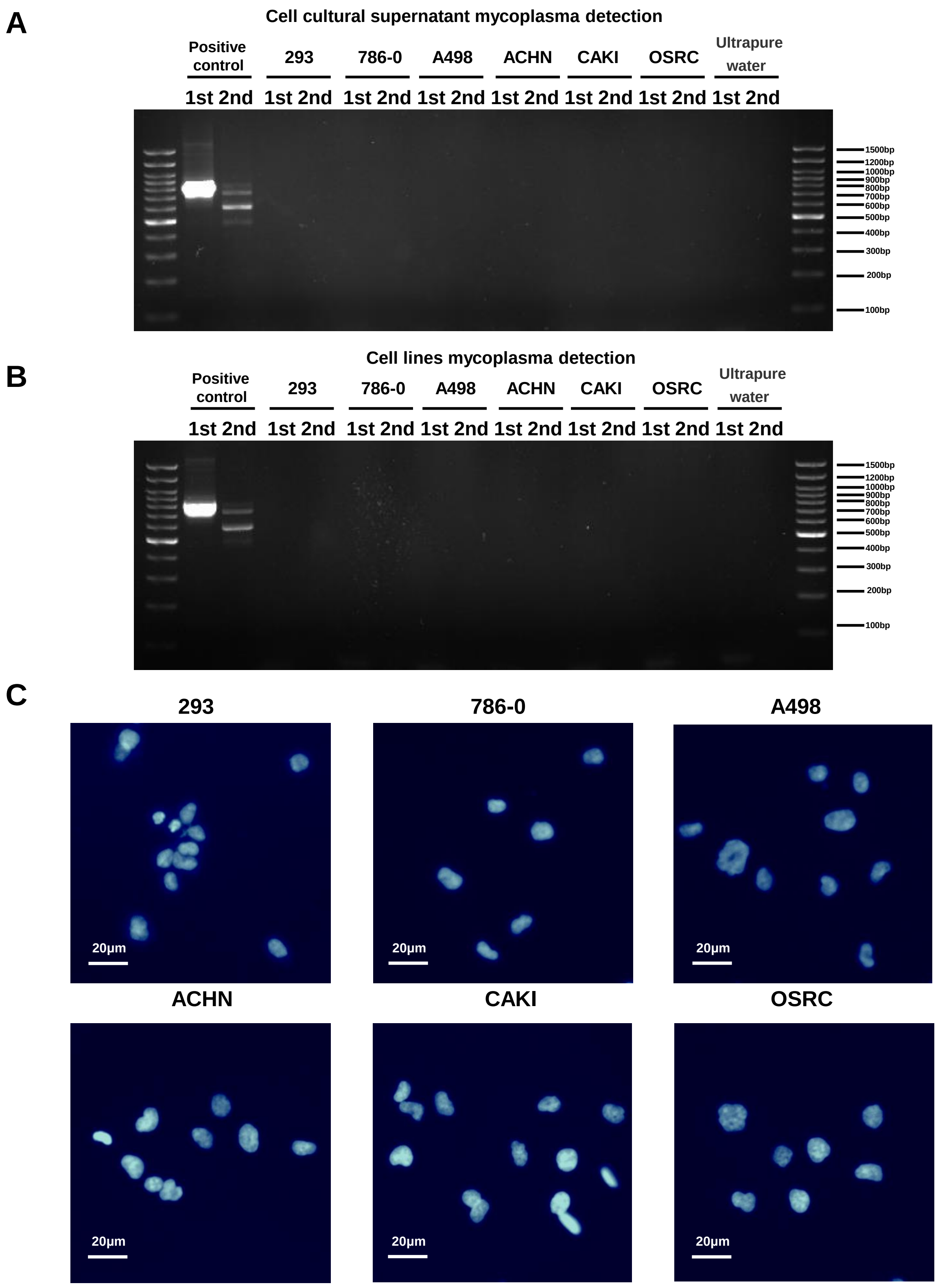

Mycoplasma detection of cell lines. (A) Cell cultural supernatant mycoplasma detection. (B) Cell lines mycoplasma detection. (C) Immunofluorescence for Mycoplasma Detection in Cell Lines.

Supporting information 2

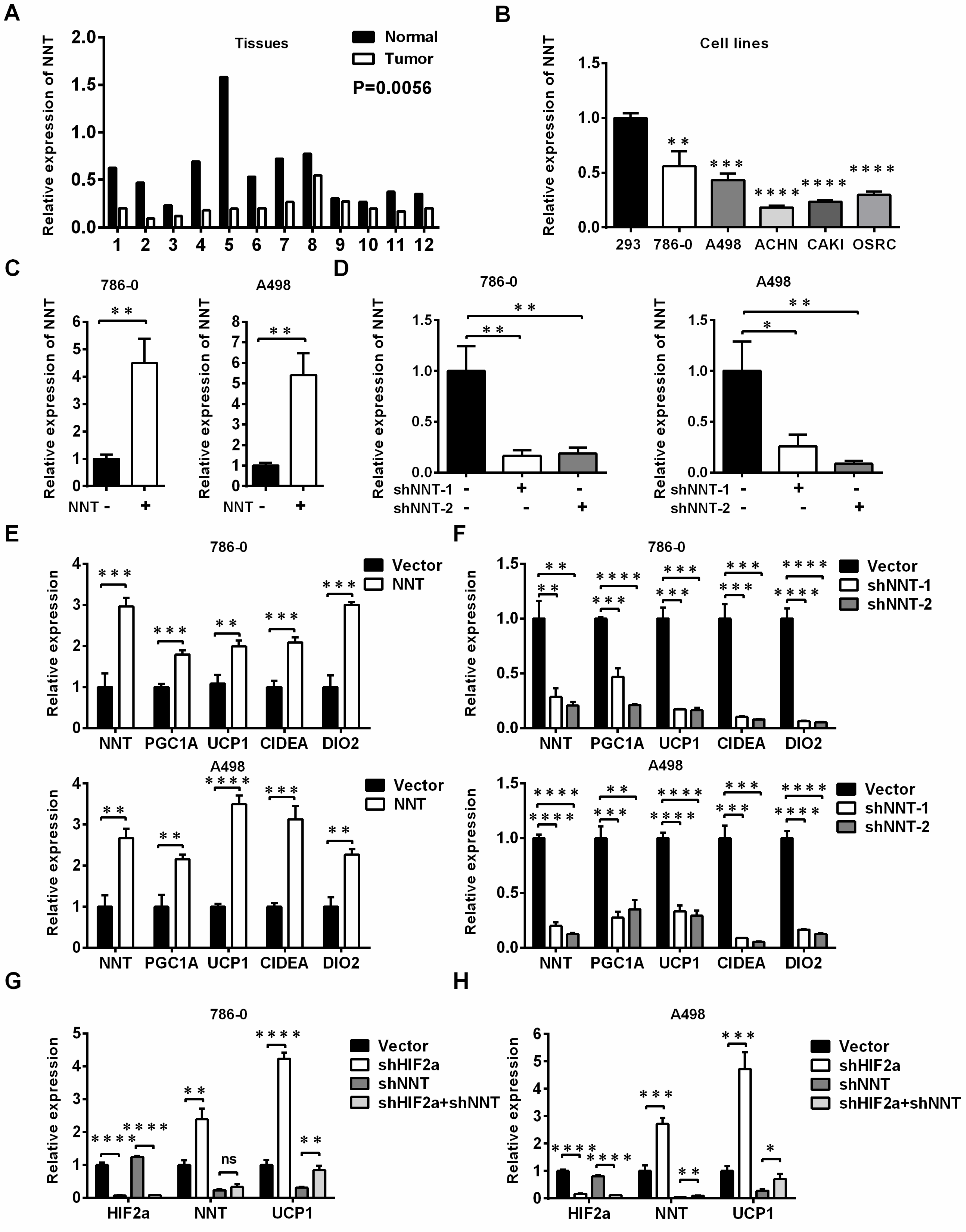

Western blot statistics. (A) Figure 1F. (B) Figure 1H. (C) Figure 2A. (D) Figure 2E. (E) Figure 4D. (F) Figure 4F. (G, H) Figure 5A.

Supporting information 3

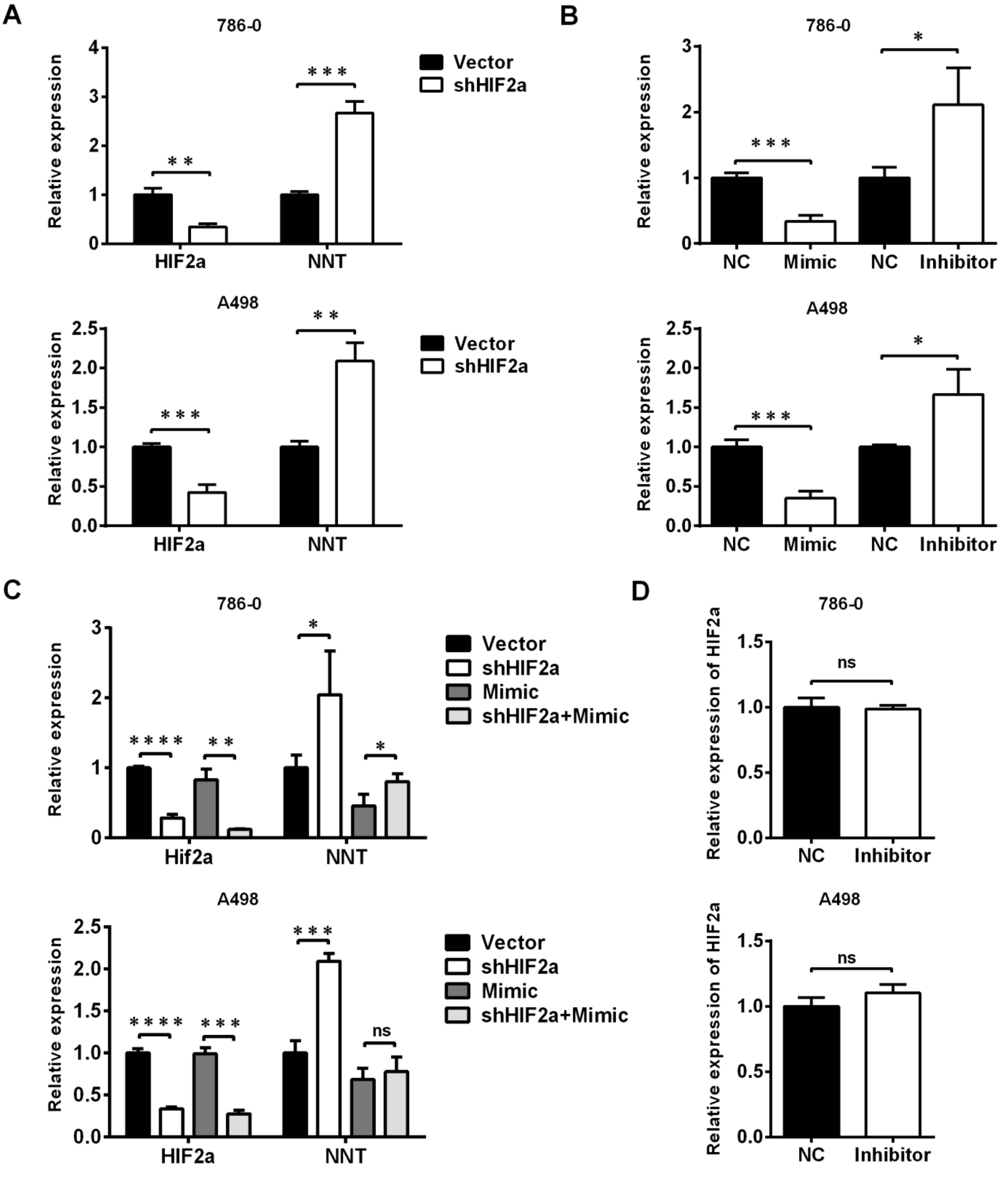

Western blot statistics. (A) Figure 6A. (B) Figure 6G. (C) Figure 6H. (D) Supplementary Figure 5D.

Supporting information 4

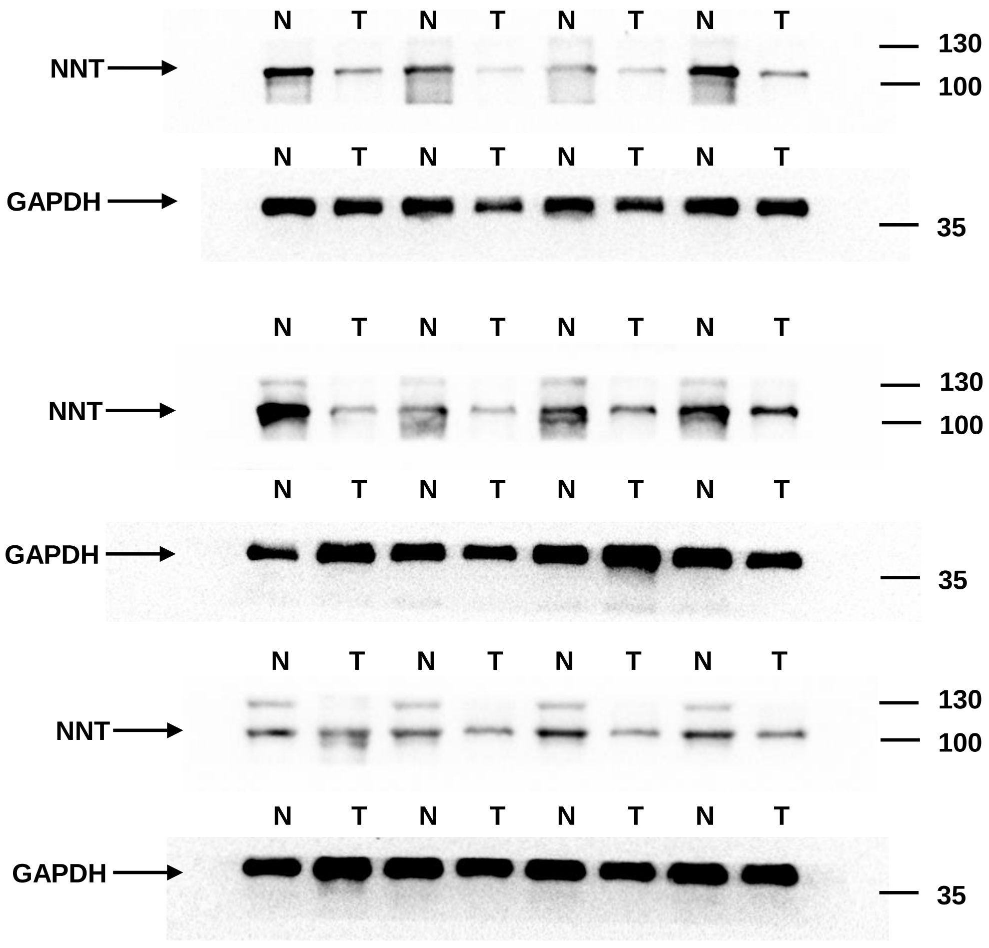

Original blots for western blots on Figure. 1F. Expression of NNT in human ccRCC tissues and paired control tissues

Supporting information 5

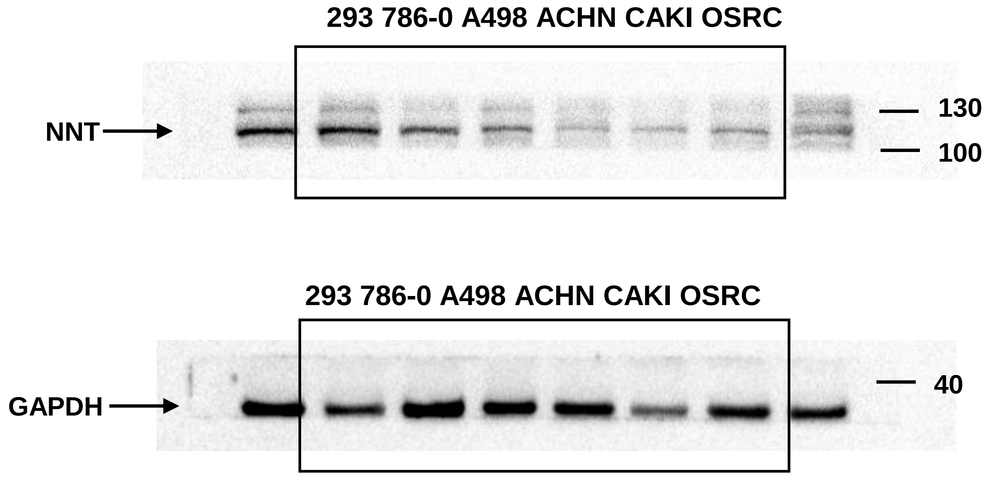

Original blots for western blots on Figure. 1H. Expression of NNT in ccRCC cell lines and control cell line

Supporting information 6

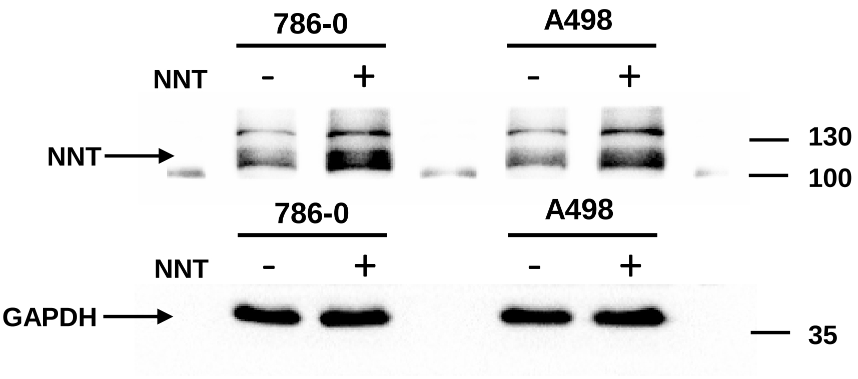

Original blots for western blots on Figure. 2A. Expression of NNT in ccRCC cell lines with NNT overexpressed.

Supporting information 7

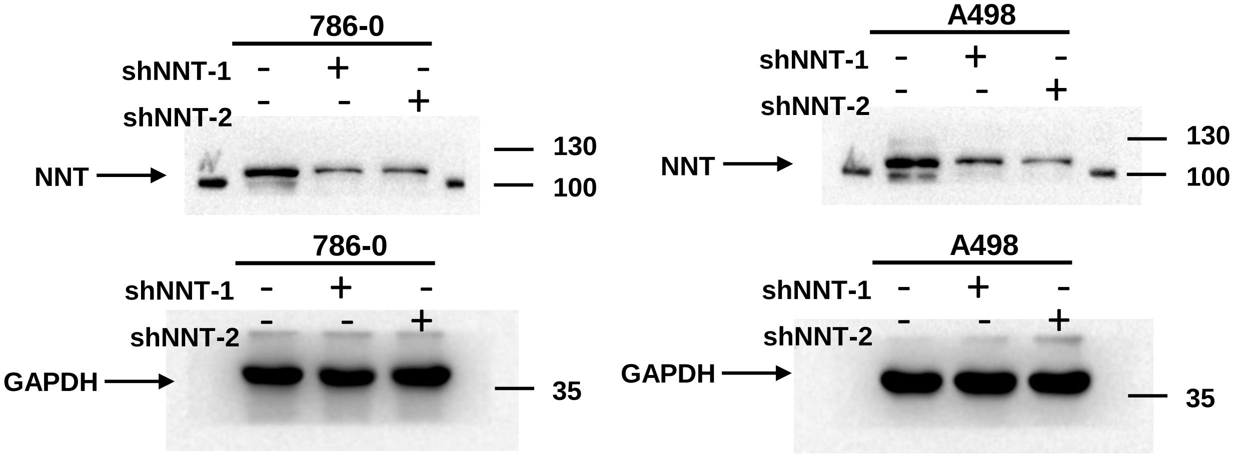

Original blots for western blots on Figure. 2E. Expression of NNT in ccRCC cell lines with NNT knocked down.

Supporting information 8

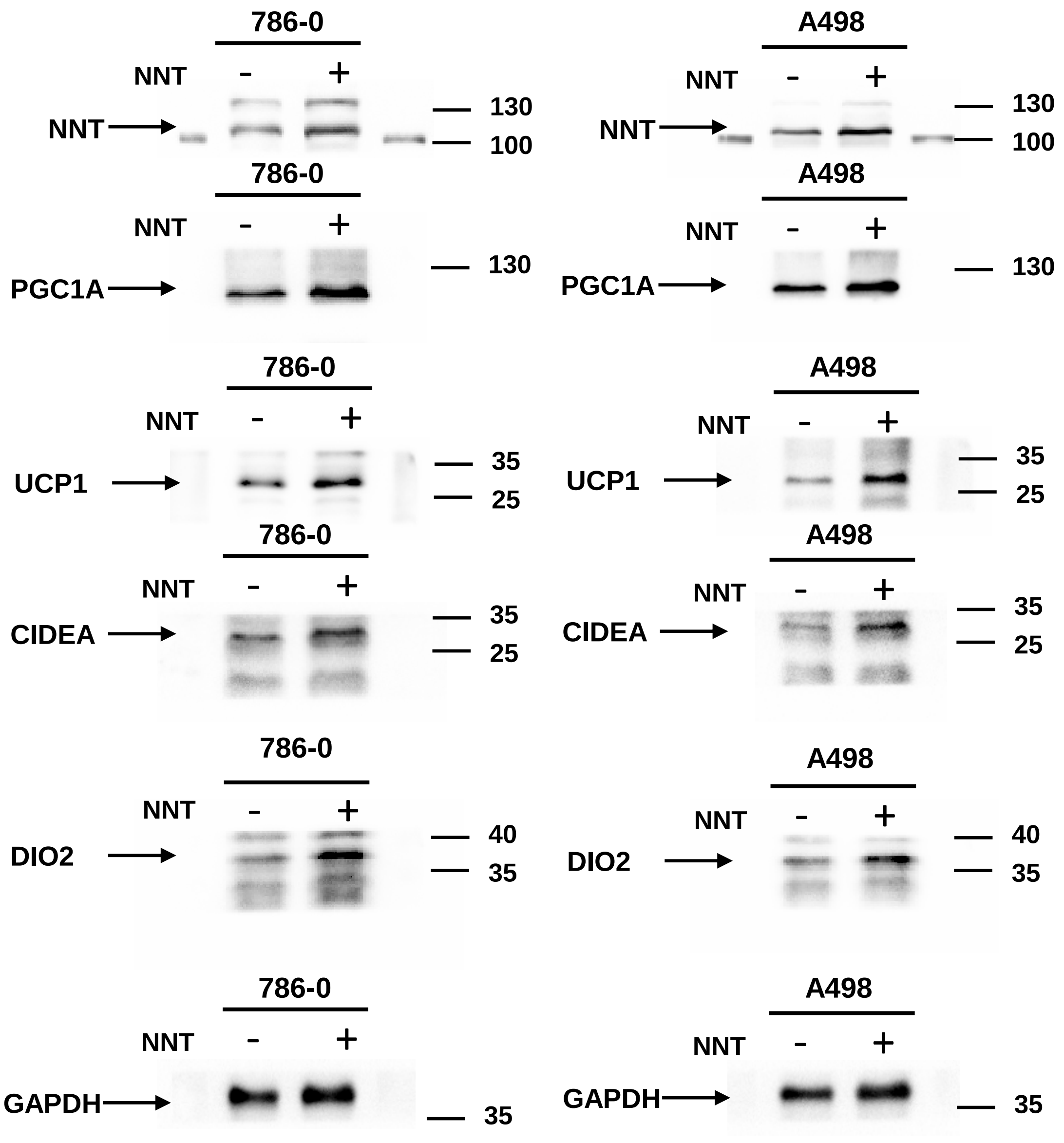

Original blots for western blots on Figure. 4D. Expression of lipid browning marker proteins in ccRCC cell lines with NNT overexpressed.

Supporting information 9

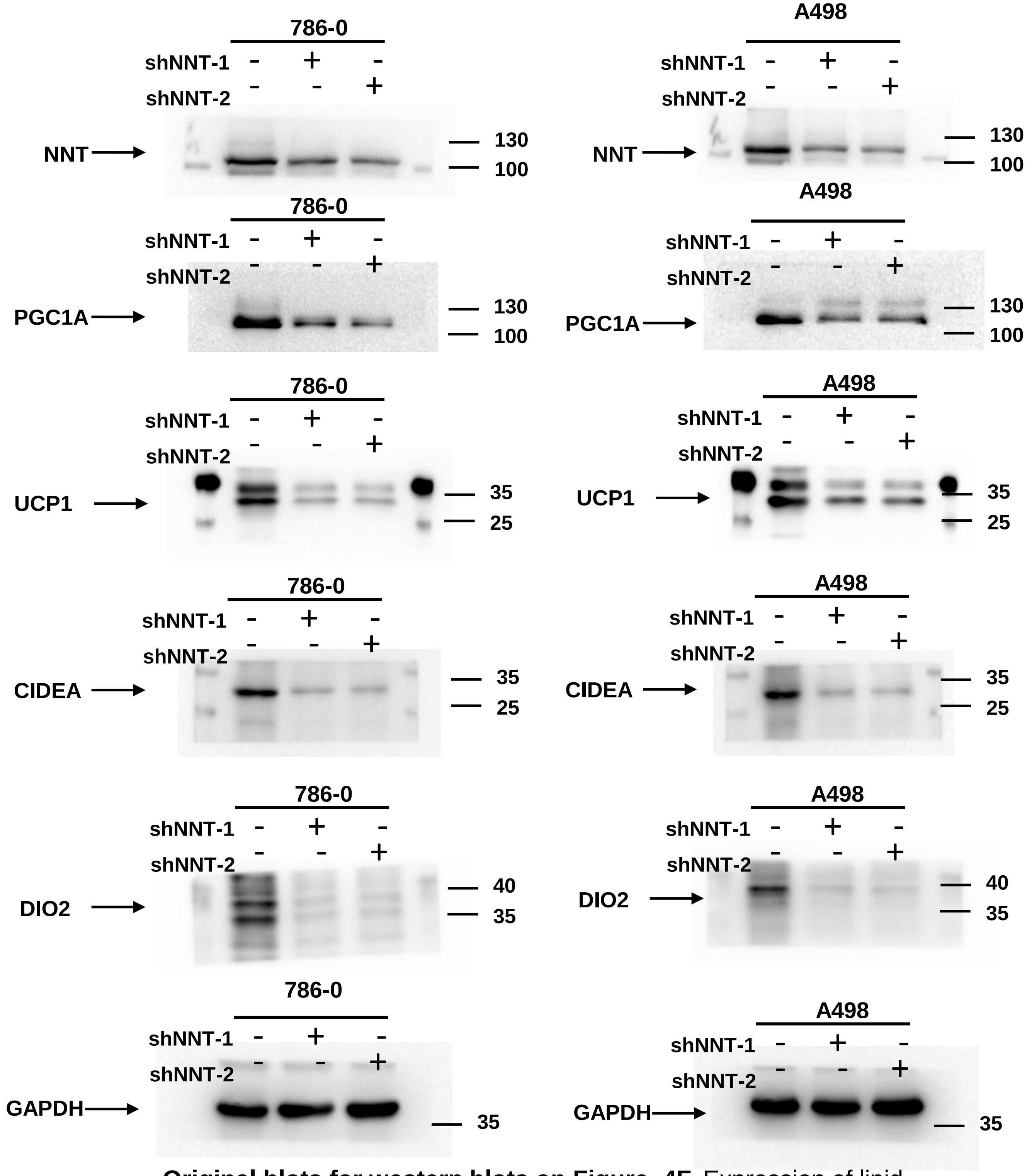

Original blots for western blots on Figure. 4F. Expression of lipid browning marker proteins in ccRCC cell lines with NNT knocked down.

Supporting information 10

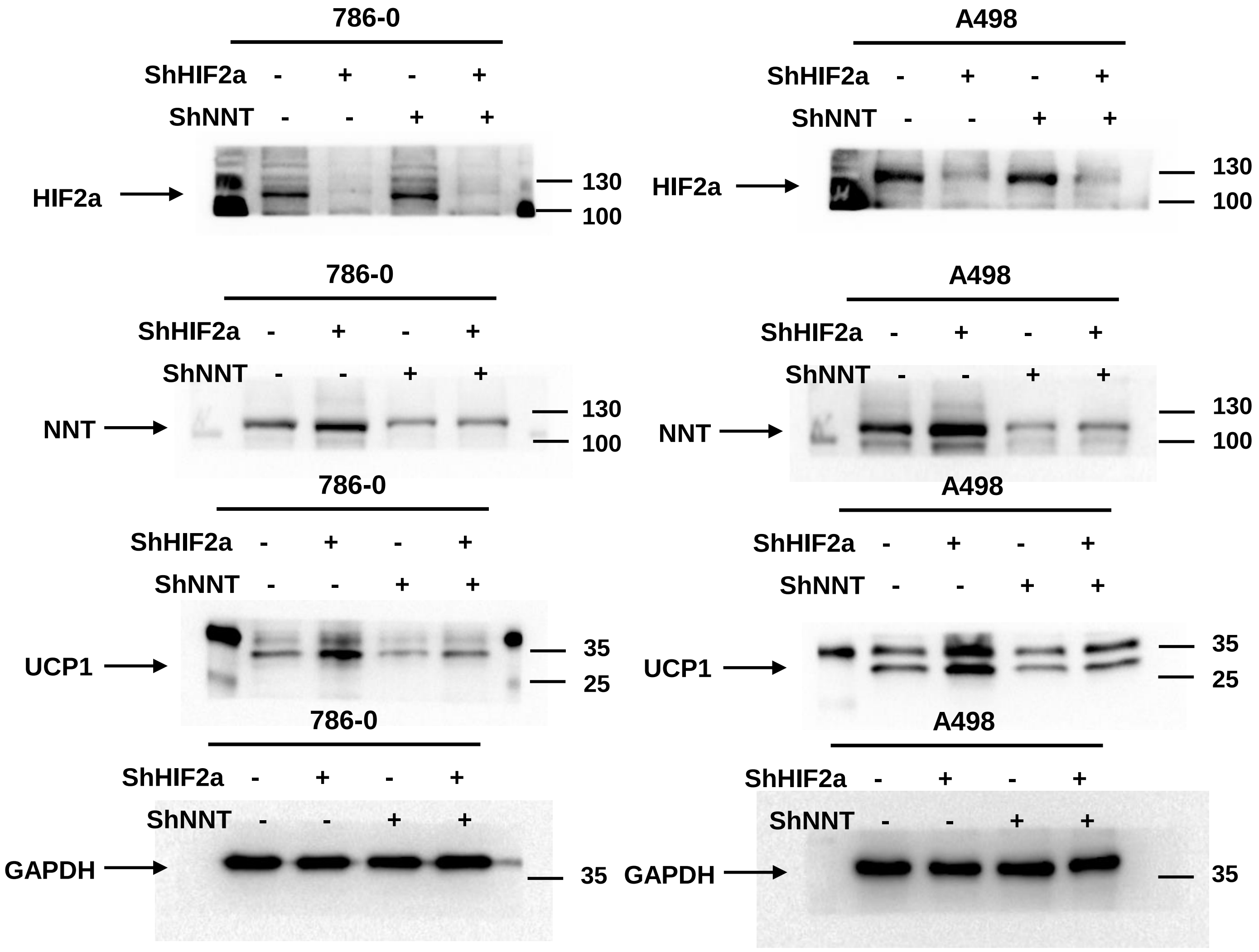

Original blots for western blots on Figure. 5A. Expression of HIF2a, NNT and UCP1 in rescue experiments.

Supporting information 11

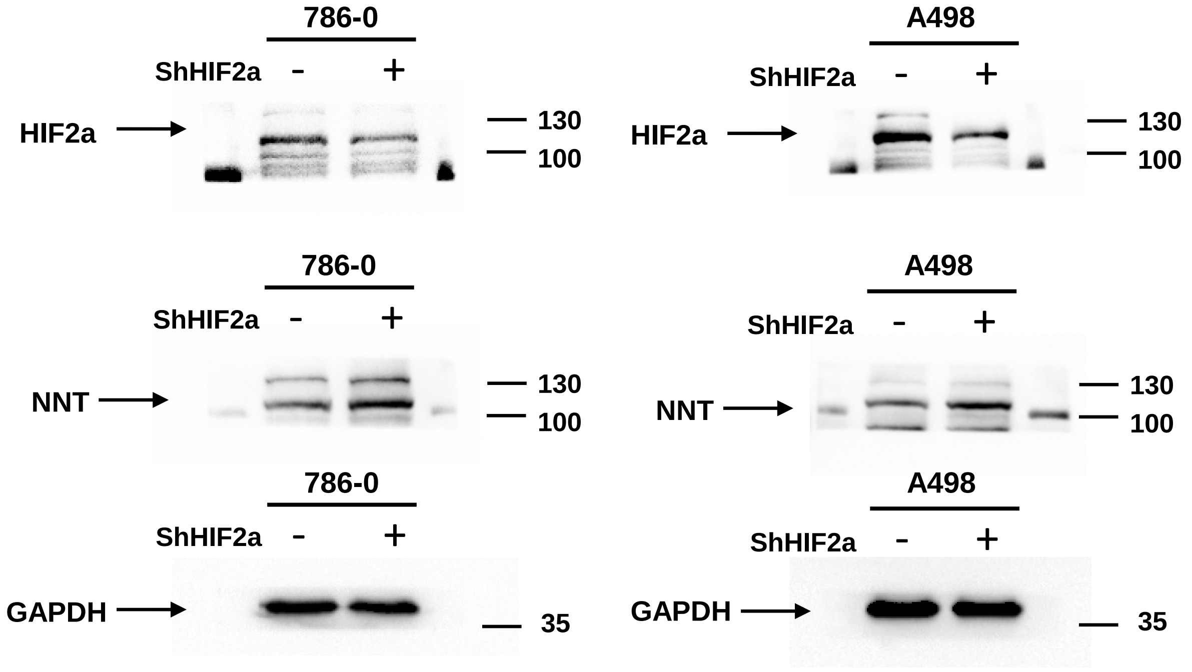

Original blots for western blots on Figure. 6A. Expression of HIF2a, NNT in cells with HIF2a knocked down.

Supporting information 12

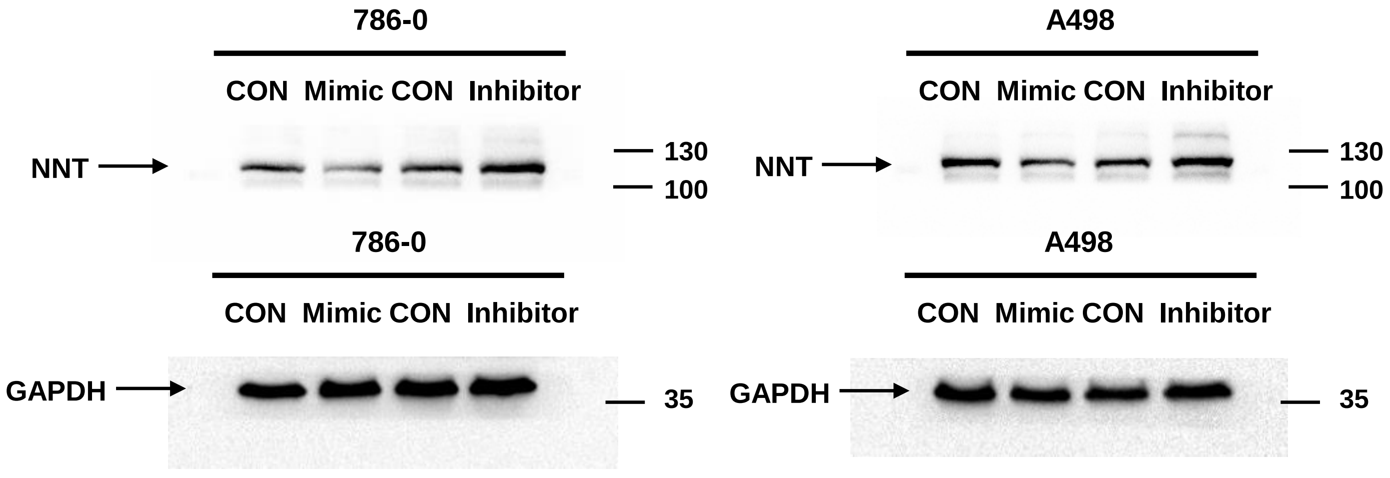

Original blots for western blots on Figure. 6G. Expression of NNT in cells treated with mimic and inhibitor.

Supporting information 13

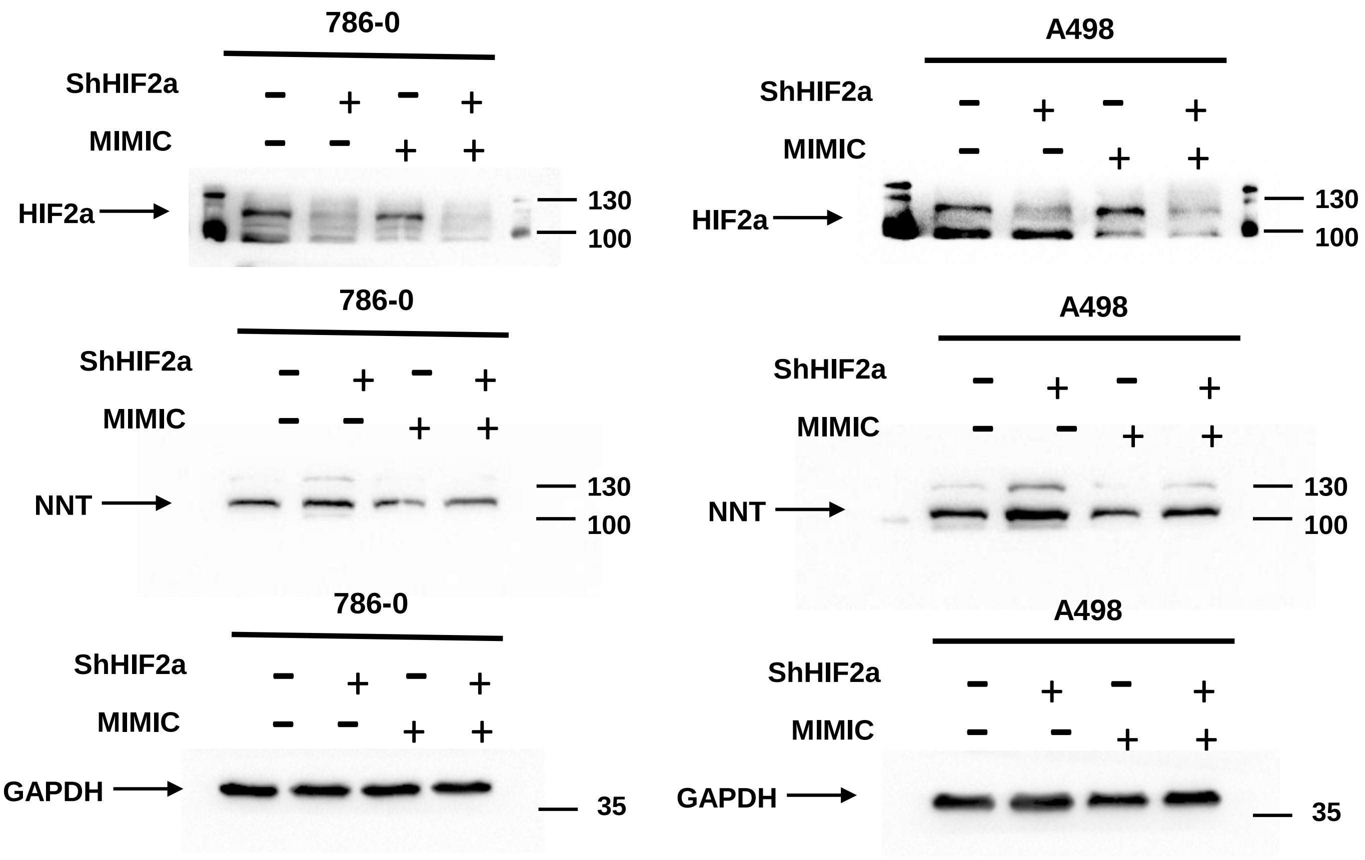

Original blots for western blots on Figure. 6H. Expression of NNT and HIF2a in rescue experiments of HIF2a and microRNA.

Supporting information 14

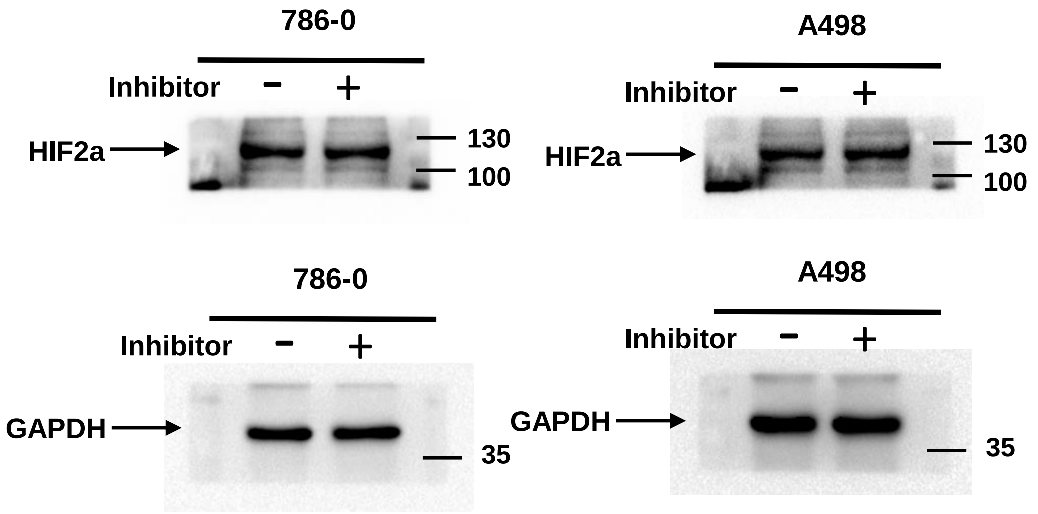

Original blots for western blots on Supplementary Figure. 5D.
Expression of HIF2a after treating cells with the miR-455-5p inhibitor.

Supporting information 15

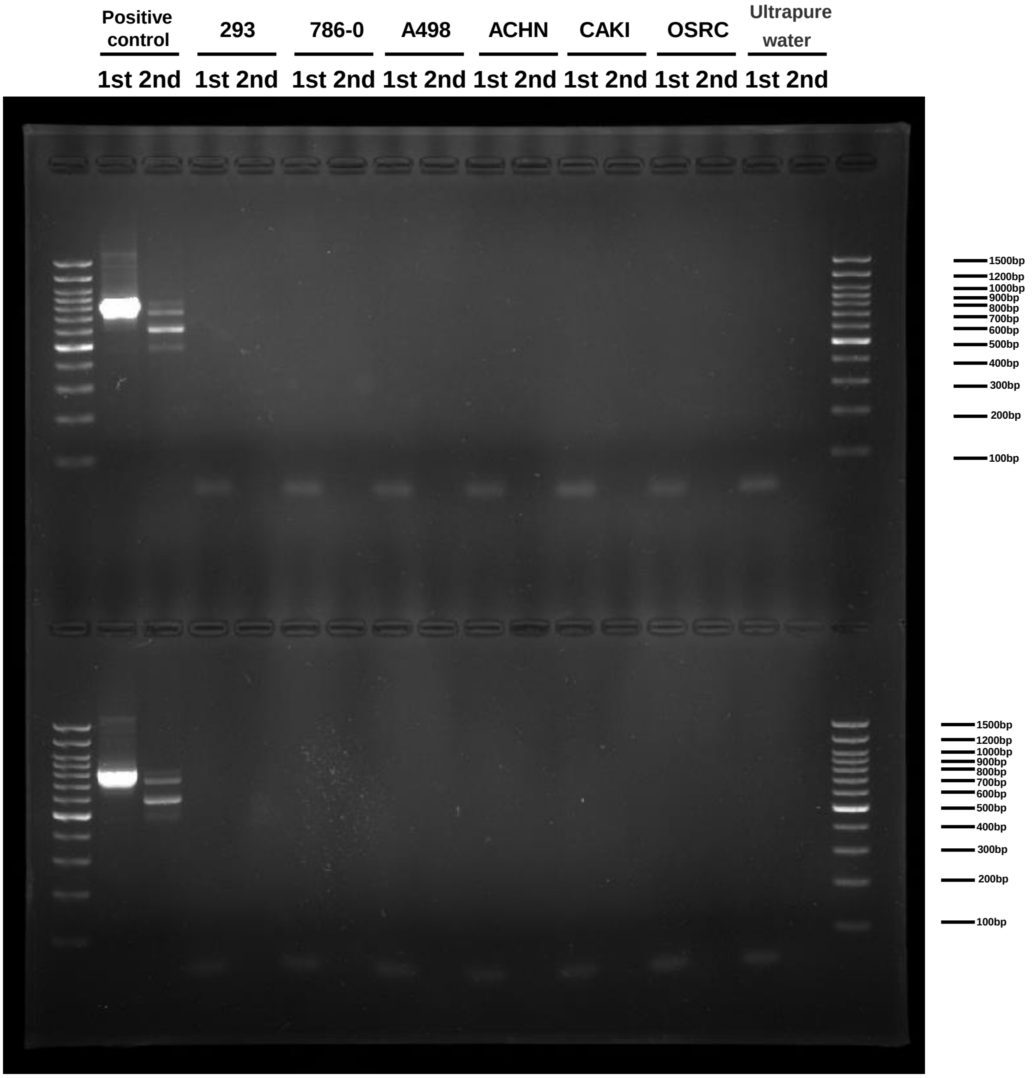

Original blots for blot on Supporting information 1. Mycoplasma detection of cell lines.
