# Supplementary material for: NNT‐induced tumor cell “slimming” reverses the pro‐carcinogenesis effect of HIF2a in tumors
Source: Clin Transl Med. 2021 Jan 12;11(1):e264. doi: 10.1002/ctm2.264 (PMC7803359; doi:10.1002/ctm2.264)
Supplement: Supplementary file 2 — Supporting Information [file CTM2-11-e264-s002.docx]

**Supplementary Table 1** Gene details of mitochondrial metabolism-related gene set

| Differential expression of mitochondrial metabolism-related genes in renal cell carcinoma | |
| --- | --- |
| \| 1. AK3 \| \| --- \| \| 1. AKAP1 \| \| 1. ARG2 \| \| 1. ATP5B \| \| 1. ATP5J \| \| 1. BCL2L13 \| \| 1. COX10 \| \| 1. COX15 \| \| 1. COX5A \| \| 1. COX7B \| \| 1. DBT \| \| 1. DECR1 \| \| 1. DLD \| \| 1. ECHDC2 \| \| 1. FH \| \| 1. GLS \| \| 1. HADHA \| \| 1. IMMT \| \| 1. ITPK1 \| \| 1. KIF1B \| \| 1. LIAS \| \| 1. MCEE \| \| 1. MRPL42 \| | 1. MRPS6 2. MUT 3. NDUFA10 4. NDUFA4 5. NDUFA5 6. NDUFAF1 7. NDUFB1 8. NDUFS1 9. NNT 10. PPIF 11. PSEN1 12. SARS2 13. SCP2 14. SIRT3 15. SLC25A29 16. SLC25A33 17. SLC25A38 18. SURF1 19. TIMM22 20. TOMM34 21. TRNT1 22. UQCRB 23. VDAC2 |

Gene set is derived from the mitochondrial metabolism project in the " differentially expressed genes in renal cell carcinoma in Higgins Renal " data subset of the Ocomine database.

**Supplementary Table 2** Gene details of mitochondrial metabolism-related gene set

| Differential expression of mitochondrial metabolism-related genes in renal cell carcinoma | | |
| --- | --- | --- |
| \| 1. ACAA2 \| \| --- \| \| 1. AGXT2L2 \| \| 1. AKAP1 \| \| 1. ALAS1 \| \| 1. ATP5A1 \| \| 1. ATP5B \| \| 1. ATP5S \| \| 1. BBC3 \| \| 1. BCAT2 \| \| 1. BCL2L1 \| \| 1. CHCHD3 \| \| 1. CKMT1B \| \| 1. COQ7 \| \| 1. COX10 \| \| 1. CS \| \| 1. DLD \| \| 1. ENDOG \| \| 1. FDX1L \| \| 1. GBAS \| \| 1. GLRX5 \| \| 1. GLS2 \| | \| 1. GOT2 \| \| --- \| \| 1. HIBADH \| \| 1. IDH3A \| \| 1. LARS2 \| \| 1. LETM1 \| \| 1. MCCC2 \| \| 1. MIPEP \| \| 1. MRPL12 \| \| 1. MRPS24 \| \| 1. MRPS25 \| \| 1. NDUFA4 \| \| 1. NDUFA5 \| \| 1. NDUFB2 \| \| 1. NDUFB9 \| \| 1. NDUFS8 \| \| 1. NDUFV1 \| \| 1. NNT \| \| 1. OGG1 \| \| 1. OXCT1 \| \| 1. PDHA1 \| \| 1. PDHB \| | 1. PDP1 2. PDPR 3. PPA2 4. PSEN1 5. SARDH 6. SLC25A15 7. SLC25A29 8. SLC25A3 9. SLC25A37 10. SLC25A39 11. SLC25A5 12. SPG7 13. SUCLG1 14. SUCLG2 15. SURF1 16. SYNJ2BP 17. TRNT1 18. TUFM 19. UQCRC1 20. UQCRC2 21. UQCRFS1 |

Gene set is derived from the mitochondrial metabolism project in the " differentially expressed genes in renal cell carcinoma in Yusenko Renal " data subset of the Ocomine database.

**Supplementary Table 3** Gene details of mitochondrial metabolism-related gene set

| Differential expression of mitochondrial metabolism-related genes in renal cell carcinoma | | | |
| --- | --- | --- | --- |
| \| 1. AASS \| \| --- \| \| 1. ACAA2 \| \| 1. ACAD8 \| \| 1. ACADS \| \| 1. ACADSB \| \| 1. ACAT1 \| \| 1. ACSS1 \| \| 1. AGMAT \| \| 1. AGXT2 \| \| 1. AIFM1 \| \| 1. ALDH1B1 \| \| 1. ALDH6A1 \| \| 1. AMT \| \| 1. ARG2 \| \| 1. ATP5D \| \| 1. ATP5G3 \| \| 1. ATP5L \| \| 1. ATP5S \| \| 1. ATPIF1 \| \| 1. AUH \| \| 1. BCKDHB \| \| 1. BCL2L1 \| \| 1. C21orf33 \| \| 1. CHDH \| \| 1. CKMT1B \| \| 1. CKMT2 \| \| 1. CPT2 \| \| 1. CRAT \| \| 1. CYP11B2 \| \| 1. CYP27B1 \| | \| 1. DHRS4 \| \| --- \| \| 1. ECHS1 \| \| 1. ETFDH \| \| 1. FDX1 \| \| 1. FECH \| \| 1. FH \| \| 1. GATM \| \| 1. GCAT \| \| 1. GCDH \| \| 1. GCSH \| \| 1. GLDC \| \| 1. GLRX5 \| \| 1. GLS \| \| 1. GLS2 \| \| 1. GLYAT \| \| 1. GOT2 \| \| 1. GSTZ1 \| \| 1. HADH \| \| 1. IDH2 \| \| 1. IDH3G \| \| 1. ISCU \| \| 1. LDHD \| \| 1. LETM1 \| \| 1. MAOA \| \| 1. ME3 \| \| 1. MIPEP \| \| 1. MOSC2 \| \| 1. MRPL34 \| \| 1. MRPL44 \| \| 1. MRPS25 \| | \| 1. MRPS28 \| \| --- \| \| 1. MUT \| \| 1. NDUFA1 \| \| 1. NDUFA11 \| \| 1. NDUFA4 \| \| 1. NDUFA6 \| \| 1. NDUFB8 \| \| 1. NDUFS1 \| \| 1. NDUFS2 \| \| 1. NDUFS8 \| \| 1. NNT \| \| 1. NUDT9 \| \| 1. OGG1 \| \| 1. OXCT1 \| \| 1. PC \| \| 1. PCCA \| \| 1. PCCB \| \| 1. PCK2 \| \| 1. PDHA1 \| \| 1. PDHB \| \| 1. PDP1 \| \| 1. PPA2 \| \| 1. PPIF \| \| 1. PPP2R1B \| \| 1. PRDX3 \| \| 1. SDHB \| \| 1. SFXN1 \| \| 1. SFXN2 \| \| 1. SFXN5 \| \| 1. SIRT3 \| | 1. SLC25A10 2. SLC25A13 3. SLC25A15 4. SLC25A16 5. SLC25A20 6. SLC25A29 7. SLC25A33 8. SLC25A35 9. SLC25A38 10. SLC25A4 11. SLC25A42 12. SLC25A5 13. SUCLG1 14. SUCLG2 15. SUOX 16. SYNJ2BP 17. TFAM 18. TIMM8A 19. TOMM40 20. TRNT1 21. TSFM 22. TXNRD2 23. UQCRB |

Gene set is derived from the mitochondrial metabolism project in the " differentially expressed genes in renal cell carcinoma in Lenburg Renal " data subset of the Ocomine database.

**Supplementary figure legends**

**Supplemental Fig. 1. NNT expression was highly correlated with the clinicopathological parameters of ccRCC.** (A) Expression of NNT in whole transcriptome sequencing data obtained after stable HIF2a knockdown. (B) The correlation heatmap and the linear correlation curve between NNT and HIF2a expression based on the data from the TCGA-KIRC database (Pearson correlation coefficient for statistics). (C) The expression of NNT in six independent additional gene sets from the Oncomine database (Independent-Samples t-test for statistics). (D) The ROC (Receiver Operating Characteristic) curves for NNT (AUC=0.9481 95% CI: 0.9099 to 0.9863; p < 0.0001) between tumor and para-tumor tissues in patients with ccRCC. (E) The Kaplan-Meier curves of NNT expression in patients with ccRCC for disease-free survival (DFS) (Log-rank for statistics). (F) The correlations between NNT expression and different clinicopathological parameters based on the data from the TCGA database (Independent-Samples t-test was used to analyze the data of two groups and ANOVA was used in the analysis of the overall expression differences of T1, T2, T3, T4 and TNM I, TNM II, TNM II, and TNM IV). Abbreviation: TCGA: The Cancer Genome Atlas; ccRCC: Clear cell renal cell carcinoma; Para: paracancerous; AUC: Area Under Curve; CI: Confidence interval; OS: Overall Survival; DFS: disease-free survival; ns: not significant; T: Tumor; N: Lymph Node; M: Metastasis.

**Supplemental Fig. 2. A low level of NNT correlated with a poor overall survival of patients with ccRCC.** The Kaplan-Meier curves of overall survival based on NNT expression were constructed for subgroups of patients with ccRCC. (A) Male. (B) Female. (C) Age ≤ 60. (D) Age > 60. (E) T1+T2 stage. (F) N0 stage. (G) Non-metastasis. (H) TNM I + II stage. (I) TNM III+ IV stage. (J) G3+G4 stage. (Log-rank for statistics). Abbreviation: T: Tumor; N: Lymph Node; M: Metastasis; G: grade.

**Supplemental Fig. 3. A low level of NNT correlated with a poor disease-free survival of patients with ccRCC.** The Kaplan-Meier analysis of ccRCC subgroups was also performed. (A) Male. (B) Female. (C) Age ≤ 60. (D) Age > 60. (E) T1+T2 stage. (F) N0 stage. (G) Non-metastasis. (H) G1+G2 stage. (I) G3+G4 stage. (Log-rank for statistics). Abbreviation: T: Tumor; N: Lymph Node; M: Metastasis; G: grade.

**Supplemental Fig. 4. NNT suppressed the migration of ccRCC cells.** (A) The migration of cells stably overexpressing NNT was analyzed by wound healing assay (Scale bar: 50μm).

**Supplemental Fig. 5. HIF2a regulated NNT expression via miR-455-5p.** (A) HIF2a mRNA level after HIF2a knockdown in ccRCC cell lines (n=3) (Independent-Samples t-test for statistics). (B) The correlation heatmap and the linear correlation curve between NNT, HIF2a and miR-455-5p, based on the data from the TCGA-KIRC database (Pearson correlation coefficient for statistics). (C) Levels of miR-455-5p in 786-0 and A498 cells treated with the miR-455-5p mimic or inhibitor (n=3) (Independent-Samples t-test for statistics). (D) HIF2 levels after treating cells with the miR-455-5p inhibitor (n=3). (E-F) Luciferase reporter assays showing that miR-455-5p overexpression significantly inhibited the activity of the wild-type NNT 3’-UTR and NNT 3’-UTR mutated at sites 2, 3, and 4 (n=3) (Independent-Samples t-test for statistics). Abbreviation: TCGA: The Cancer Genome Atlas; KIRC: kidney renal clear cell carcinoma; ccRCC: Clear cell renal cell carcinoma; NC: Negative control; has: homo sapiens; WT: wild type; mut: mutant.

**Supplemental Fig. 6. The potential binding sites for HIF2a in the miR-455-5p promoter.** (A) Based on the HIF2a binding sequence, three potential binding sites were identified in the 2000 bp region upstream of the miR-455-5p promoter: sites A, B, and C.

**Supplemental Fig. 7.** **The specific construction sequences of truncated plasmid.** (A) The specific construction sequences of truncated plasmid

Supplementary Fig1





Supplementary Fig2





Supplementary Fig3





Supplementary Fig4





Supplementary Fig5


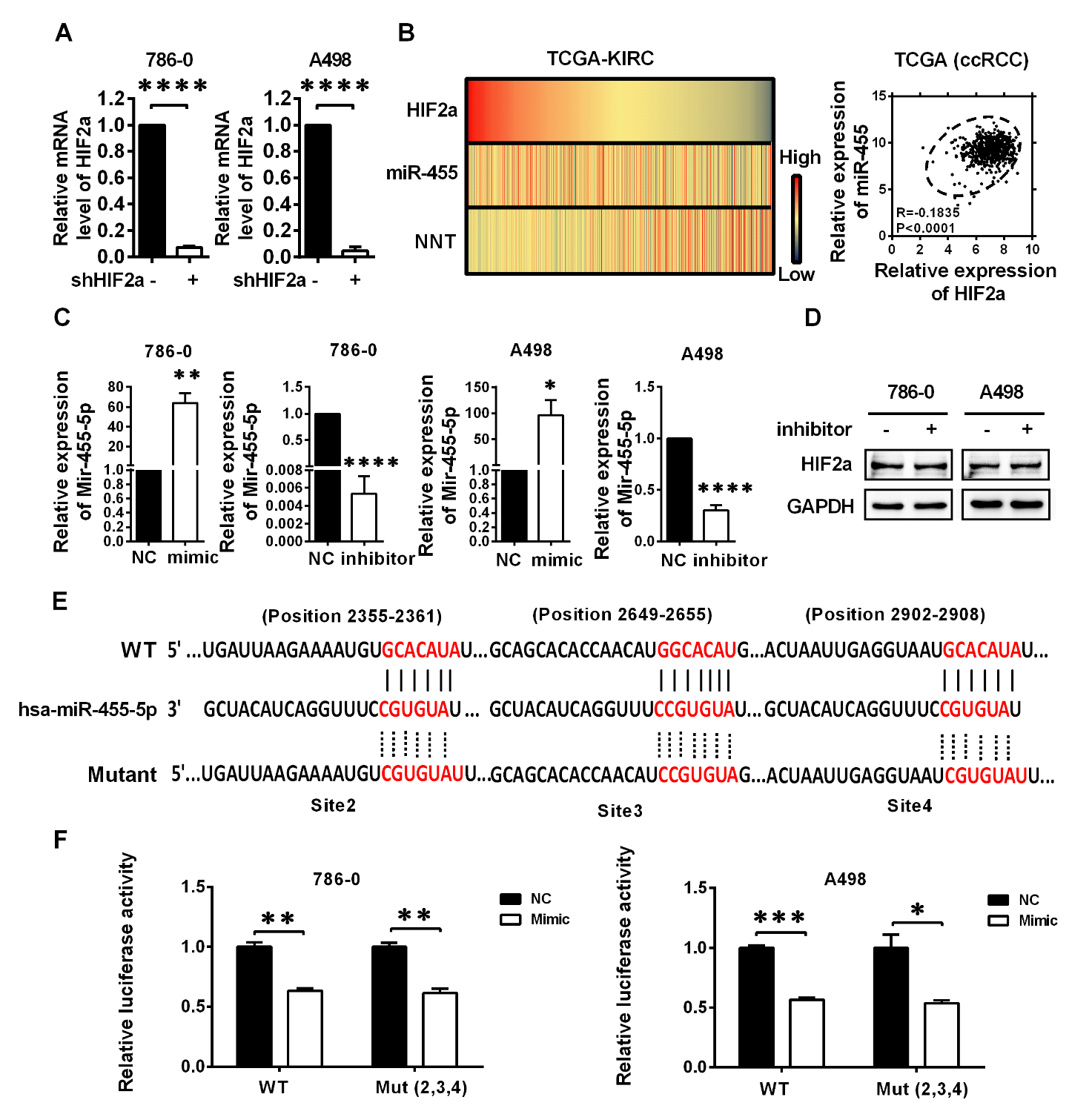


Supplementary Fig6





Supplementary Fig7
